# Antioxidant properties of butylated phenol with oxadiazole and hydrazone moiety at *ortho* position supported by DFT study†

**DOI:** 10.1039/d2ra02140d

**Published:** 2022-06-09

**Authors:** Raied M. Shakira, Muhammad Kumayl Abd Wahab, Nurdiana Nordin, Azhar Ariffin

**Affiliations:** Department of Chemistry, Faculty of Science, Universiti Malaya 50603 Kuala Lumpur Malaysia ndiana13@um.edu.my azhar70@um.edu.my +60 7967 4193 +60 7967 7022 +60 7967 4080; Department of Chemistry, Ibn Al-Haitham University of Baghdad Baghdad Iraq

## Abstract

Two series of 1,3,4-oxadiazole derivatives at the sixth position of the 2,4-di-*tert*-butylphenol group were synthesized. The antioxidant properties were evaluated by DPPH and FRAP assays. Compound 3 showed significant antioxidant activity, while its alkyl derivatives exhibited decreased antioxidant activity in both assays. The preferential antioxidant mechanism of the reactive antioxidant molecules prepared from the further reaction of compound 3 to produce compounds 4 and 6 was investigated using density functional theory. Calculating their comprehensive reactivity descriptors was used to assess their antioxidant reactivity. According to the calculated descriptors, compounds 4c and 6d are the most reactive antioxidants within their own group compared to the other derivative moieties. The results are identical to ascorbic acid's, indicating that they have similar activity. The experimental data and the calculated descriptors are in good agreement. The nature of the substituents and their positions have a significant impact on the derivatives' antioxidant capabilities.

## Abstract 2

Two series of 1,3,4-oxadiazole derivatives at the sixth position of the 2,4-di-*tert*-butylphenol group were synthesized.

## Graphical abstract

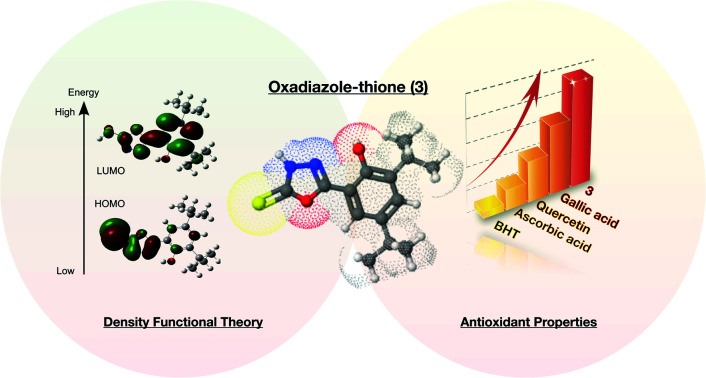

## Introduction

Excessive production of reactive free radicals in the body, which are produced by a variety of enzymatic and nonenzymatic activities, causes substantial macromolecule damage in protein, DNA, membrane lipids, and carbohydrates, resulting in cellular damage. The oxidative stress that results is thought to play a role in the ageing process and the development of degenerative diseases such as type 2 diabetes, inflammation, neurological diseases, and malignancies.^1–3^ As a result, there is a growing interest in using medicinal substances with antioxidant activity to block these unwanted oxidative events by trapping free-radical intermediates produced during oxidative reactions.^4–7^

Recently, many reports have emphasised that phenol derivatives play a very important role in human health and in therapeutics,^8–10^ as well as in industry,^11^ where they are employed as stabilisers,^12–14^*via* their antioxidant activity. Hindered phenols are notably interesting antioxidant compounds,^15,16^ in which the di-*tert*-butylphenol can be considered a crucial starting material in preparing antioxidant compounds.^17,18^ Many derivatives of the di-*tert*-butylphenol group (containing a heterocyclic group) have been observed to show good biological activity (in addition to antioxidant activity), such as anti-inflammatory activity,^19^ the growth inhibition of lung cancer^20^ and the development of cyclooxygenase-2 inhibitors.^21^ This antioxidant activity is not only confined to di-*tert*-butyl groups flanking the hydroxyl group (such as 2,6-DTBP) but also the semi-hindered phenol with one *tert*-butyl group bonded to the hydroxyl in position 2 and the other at position 4.^22,23^

Conversely, 2,4-di-*tert*-butylphenol is known as an antioxidant, and it has been reported that its antioxidant activity is lower than that of BHT (butylated hydroxytoluene) and that the *tert*-butyl group in the *para* position can reduce the compound's antioxidant ability.^24^ Furthermore, compounds that can be classified as strong antioxidants usually share common structural features. For example, such compounds often have a hindered phenol^25^ or multiple phenolic hydroxyl groups, *e.g.*, flavonoids,^26,27^ or have a fully conjugated π system, *e.g.*, carotenoids.^28^ It is interesting to note that substituted groups may also influence the scavenging ability of such compounds, as mentioned previously, which indicates the existence of a close relationship between chemical structure and the ability to scavenge free radicals. The 1,3,4-oxadiazoles and their 2,5-disubstituted derivatives function well as development materials due to their large spectrum of various biological activities, such as anti-inflammatory,^29^ anticancer,^30^ antibacterial,^31^ antifungal,^18^ antihypertensive,^32^ antitubercular,^33^ anti-HIV^34^ and antioxidant activities.^35^ Moreover, some oxadiazoles derivatives have exhibited antioxidant activities even though they do not contain any phenol group.^36^

**Fig. 1:**
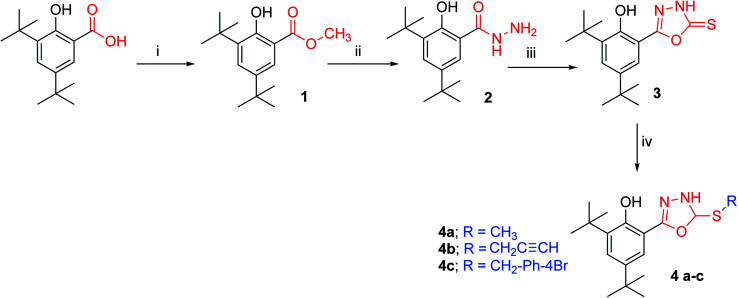
Reaction pathway for the synthesis of compounds 3 and 4a–c (i) MeI, NaHCO3, DMF, reflux 9 h. (ii) Heating to melting point, N_2_H_4_·H_2_O. (iii) CS_2_, KOH, EtOH, reflux 3 h. (vi) RX, K_2_CO_3_, acetone, r.t.

**Fig. 2:**
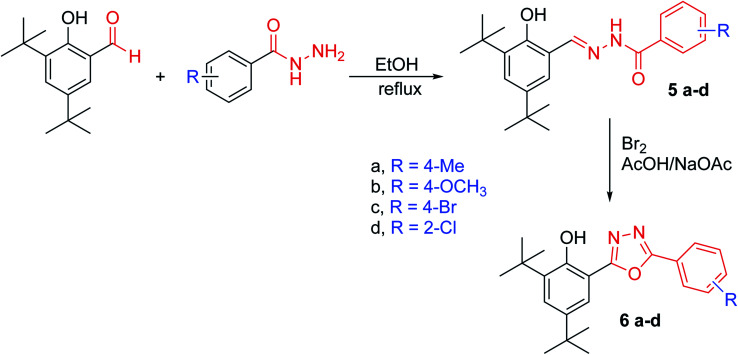
Reaction pathway for the synthesis of compounds 5a–d and 6a–d.

In this study, we synthesised a 1,3,4-oxadiazole ring at position six of 2,4-di-*tert*-butyl phenol. We aim to accomplish the phenol's antioxidant activity (to be approaching that of BHT or slightly less) by increasing the steric hindrances surrounding its hydroxyl while increasing free radical stability (which is formed after the phenol donates its hydrogen atom). Hydrazones of 2,4-DTBP (as an intermediate in the formation the oxadiazole ring) have also been investigated for their antioxidant ability. The synthesised compounds are illustrated in Fig. 1 and 2. In addition, we performed a systematic analysis of seven synthesised compounds of 1,3,4-oxadiazole ring at position six of 2,4-di-*tert*-butyl phenol derivatives by means of structural conformational and electron analyses of protonated (neutral) and deprotonated forms. In the context of conceptual DFT, the global reactivity descriptors of neutral compounds have been derived. These calculations may result in the implementation of biological antioxidants by illustrating the SAR and radical scavenging mechanisms of these naturally occurring chemicals.

## Experimental section

### Materials

Unless otherwise noted, all materials were purchased from commercial suppliers and used without purification. The melting points of the materials were determined by the open capillary tube method using MEL-TEMP II apparatus and were uncorrected. The purities of compounds were verified by thin layer chromatography (silica gel TLC) using plates from Merck, and spots were located under iodine vapour and UV radiation. IR spectra were recorded using a PerkinElmer 400 Fourier Transform Infrared (FTIR) Spectrometer. NMR spectra were recorded on a JEOL-ECA 400 MHz, JEOL-Lambda 400 or Bruker 400AVN MHz spectrometer, with CDCl_3_ and DMSO-*d*_6_ used as the solvent and TMS as the internal standard. Mass spectra were recorded using an Agilent 5975 for EI/MS and Finnigan TSQ7000 for HREI/MS (NUS Singapore). UV spectroscopy Power Wave X340 (BIO-TEK Instruments, Inc.) was used to record the FRAP and DPPH assays.

### Synthesis of methyl 3,5-di-*tert*-butyl-salicylate (1)

A 100 mL round-bottom flask was charged with 3,5-di-*tert*-butyl-salicylic acid (3.75 g, 15 mmol) and sodium hydrogen carbonate (1.3 g, 15.5 mmol) in 20 mL dry DMF. Excess methyl iodide was added. The mixture was refluxed for 9 h, and the reaction was monitored by TLC using a hexane : ethyl acetate solution mixed in a 5 : 1 ratio. Excess solvent was evaporated under reduced pressure. 25 mL distilled water was added to the solid residue and extracted with 50 mL ethyl acetate. The organic layer was washed with water and dried over anhydrous magnesium sulphate. The solvent was evaporated under reduced pressure. The crude product was recrystallised from ethanol–hexane to afford a pure white solid ester. Yield 3.76 g (94%); mp 72–74 °C; *ν*_max_ (KBr)/cm^−1^: 3105 (CH_aromatic_), 2957, 2908, 2870 (CH_aliphatic_), 1727 (C=O) 1600, 1559 (C=C), 1215 (C–O). ^1^H NMR (CDCl_3_) *δ* 1.28 (s, 9H, *t*-Bu), 1.41 (s, 9H, *t*-Bu), 3.92 (s, 3H, OCH_3_), 7.57 (d, 1H, *J* 2.76), 7.78 (d, 1H, *J* 2.76), 11.32 (s, 1H, OH). ^13^C NMR (CDCl_3_) *δ* 29.47 (3C), 31.50 (3C), 34.38 (C), 35.25 (C), 52.28 (C, OCH_3_), 110.50 (C), 124.69 (C), 131 (C), 137.51 (C), 140.96 (C), 159.79 (C), 176.05 (C, CO). HREIMS found 264.1725 [M˙+] (calc. for C_16_H_24_O_3_, 264.1725).

### Synthesis of 3,5-di-*tert*-butyl-salicylic hydrazide (2)

Methyl 3,5-di-*tert*-butyl-salicylate (2.64 g, 10 mmol) was heated to its melting point in a 50 mL round-bottom flask. When the ester has melted, 5 mL of hydrazine hydrate was added dropwise, and the mixture was heated to 70–75 °C for 2 h. Absolute ethanol was added until a clear solution appeared and then refluxed for another 3 h. Upon cooling, the white precipitate was filtered and washed. Recrystallisation of the crude product from aqueous ethanol afforded a white precipitate. Yield 2.53 g (96%); mp 192–194 °C; *ν*_max_ (KBr)/cm^−1^: 3675 (OH_phenol_), 3316, 3208, 3192 (NHNH_2_), 2960, 2871 (CH_aliphatic_), 1626 (C=O), 1593 (C=C). ^1^H NMR (DMSO-*d*_6_) *δ* 1.26 (s, 9H, *t*-Bu), 1.47 (9H, s, *t*-Bu), 4.93 (brs, 2H, NH_2_), 7.35 (1H, s), 7.83 (s, 1H), 10.28 (brs, 1H, NH), 13.40 (brs, 1H, OH). ^13^C NMR (DMSO-*d*_6_) *δ* 29.68 (3C), 31.79 (3C), 34.61 (C), 35.14 (C), 112.82 (C), 121.21 (C), 127.92 (C), 136.83 (C), 140.22 (C), 157.82 (C), 170.11 (C). HREIMS found 264.1838 [M˙^+^] (calc. for C_15_H_24_O_2_N_2_, 264.1838).

### Synthesis of 2,4-di-*tert*-butyl-6-(5-thio-4-hydro-1,3,4-oxadiazol-2-yl)phenol (3)

To a solution of hydrazide (1 g, 3.75 mmol) and excess carbon disulphide (0.6 mL) in absolute ethanol, potassium hydroxide (0.21 g, 3.75 mmol) was added in one portion at ambient temperatures. The mixture was stirred and refluxed for 3 h, and the solvent was removed under vacuum. Distilled water (25 mL) was added to the residue and stirred for another 15 minutes. The residue was filtered, and the filtrate was acidified with 5% hydrochloric acid and then filtered once more. The white precipitate was washed with water and recrystallised with ethanol. Yield 0.91 g (79%); mp 222–224 °C; *ν*_max_ (KBr)/cm^−1^: 3187 (bs, OH, NH), 3092 (CH_aromatic_), 2956 (CH_aliphatic_), 1618 (C=N), 1594, 1580 (C=C), 1270 (C=S), 1218 (C–O), 1095 (C–O–C). ^1^H NMR (CDCl_3_) *δ* 1.27 (s, 9H, *t*-Bu), 1.38 (s, 9H, *t*-Bu), 7.41 (d, 1H, *J* 2.23), 7.45 (d, 1H, *J* 2.20), 9.1 (brs, 1H, OH). ^13^C NMR (CDCl_3_) *δ* 29.85 (3C), 31.66 (3C), 34.61 (C), 35.48 (C), 109.48 (C), 122.13 (C), 128.34 (C), 137.88 (C), 142.35 (C), 152.96 (C), 161.11 (C, C=N), 177.10 (C, C=S). HREIMS found 306.1402 [M˙^+^] (calc. for C_16_H_22_N_2_O_2_S, 306.1402).

### General alkylation 2,4-di-*tert*-butyl-6-(5-thio-4-hydro-1,3,4-oxadiazol-2-yl) phenol (4a–c)

Alkyl halide (1 mmol) was added in small portions to a stirred suspension of 1,3,4-oxadiazol (0.31 g, 1 mmol) in dry acetone and anhydrous potassium carbonate (0.14 g, 1 mmol). The mixture was left to stir overnight at ambient temperature. The solvent was evaporated and the residue extracted with 25 mL chloroform. The residue was dried over anhydrous magnesium sulphate and recrystallised from a suitable solvent.

#### 2,4-Di-*tert*-butyl-6-(5-methylthio-1,3,4-oxadiazol-2-yl)phenol (4a)

The crude product was recrystallised from methanol to afford white needle-like crystals. Yield 0.26 g (83%); mp 100–102 °C; *ν*_max_ (KBr)/cm^−1^: 3163 (br, OH), 2950, 2868, 1614 (C=N), 1595, 1482 (C=C), 1178 (C–O), 1095 (C–O–C). ^1^H NMR (CDCl_3_) *δ* 1.31 (s, 9H, *t*-Bu), 1.44 (s, 9H, *t*-Bu), 2.78 (s, 3H, SCH_3_), 7.46 (d, 1H, *J* 2.44), 7.52 (d, 1H, *J* 2.44), 10.21 (brs, 1H, OH). ^13^C NMR (CDCl_3_) *δ* 14.77 (C, SCH_3_), 29.48 (3C), 31.53 (3C), 34.46 (C), 35.39 (C), 107.47 (C), 120.64 (C), 128.46 (C), 137.46 (C), 141.79 (C), 154.30 (C), 164.12 & 166.35 (C7, C8, C=N). HREIMS found 320.1562 [M˙^+^] (calc. for C_17_H_24_N_2_O_2_S, 320.1558).

#### 2,4-Di-*tert*-butyl-6-(5-prop-2-yn-1-ylthio-1,3,4-oxadiazol-2-yl)phenol (4b)

The crude product was recrystallised from ethanol to afford white crystals. Yield 0.3 g (86%); mp 108–110 °C; *ν*_max_ (KBr)/cm^−1^, 3276 (C≡CH), 3153(OH_phenol_), 2951, 2868 (CH_aliphatic_), 2164(C≡C), 1617 (C=N), 1995, 1557 (C=C), 1178 (C–O), 1095 (C–O–C); ^1^H NMR (DMSO-*d*_6_) *δ* 1.29 (s, 9H, *t*-Bu), 1.41 (s, 9H, *t*-Bu), 3.37 (t, 1H, *J* 2.67), 4.23 (d, 2H, *J* 2.68), 7.48 (d, 1H, *J* 2.44), 7.59 (d, 1H, *J* 2.44), 10.18 (brs, 1H, OH). ^13^C NMR (CDCl_3_) *δ* 20.95 (SCH_2_ C≡), 29.21 (3C), 31.09 (3C), 34.06 (C), 34.88 (C)75.16 ((≡CH)CH), 78.92 (C≡CH), 107.69 (C), 121.13 (C), 128.16 (C), 136.90 (C), 141.90 (C4), 153.30 (C), 161.98 & 166.00 (C7 & C8). HREIMS found 344.1558 [M˙^+^] (calc. for C_19_H_24_N_2_O_2_S, 344.1558).

#### 2,4-Di-*tert*-butyl-6-(5-(4-bromobenzyl)thio-1,3,4-oxadiazol-2-yl)phenol (4c)

The solid product was recrystallised from ethanol-ethyl acetate to afford a white amorphous solid. Yield 0.37 g (78%); mp 118–120 °C; *ν*_max_ (KBr)/cm^−1^: 3178 (OH_phenol_), 3045 (CH_aromatic_), 2958, 2845 (CH_aliphatic_), 1616 (C=N), 1995, 1845 (C=C), 1265 (C–O), 1081 (C–O–C). ^1^H NMR (CDCl_3_) *δ* 1.32 (s, 9H, *t*-Bu), 1.45 (s, 9H, *t*-Bu), 4.46 (s, 2H), 7.35 (d, 2H, *J* 8.41), 7.36–7.50 (m, 4H). ^13^C NMR (CDCl_3_) *δ* 29.39 (3C), 31.44 (3C), 34.38 (C), 35.32 (C), 36.16 (C), 107.27 (C), 120.57 (C), 122.26 (C), 128.52 (C), 130.76 (2C), 131.99 (2C), 134.64 (C), 137.43 (C), 141.75 (C), 154.26 (C), 162.38 & 166.40 (2C, C=N). HREIMS found 474.0960 [M˙^+^] (calc. for C_23_H_27_BrN_2_O_2_S, 474.0956).

### General synthesis of *N*′-[(3,5-di-*tert*-butyl-2-hydroxyphenyl)methylidene]- substituted benzohydrazide (5a–d)

To a warm stirring solution of aryl hydrazide (3 mmol) in 20 mL absolute ethanol, 3,5-di-*tert*-butyl-salicylaldehyde (0.70 g, 3 mmol) was added in small portions and refluxed for 7 h. Upon cooling, the mixture was stored overnight in a refrigerator at 5 °C. The precipitate was washed with cold ethanol and recrystallised from a suitable solvent.

#### *N*′-[(3,5-Di-*tert*-butyl-2-hydroxyphenyl)methylidene]-4-methylbenzohydrazide (5a)

The crude product was recrystallised from ethanol to afford a white precipitate. Yield 0.95 g (87%); mp 314–316 °C; *ν*_max_ (KBr)/cm^−1^: 3675 (OH_phenol_), 3160 (NH), 3090 (CH_aromatic_), 2955, 2868 (CH_aliphatic_), 1651 (C=O), 1611 (C=N), 1595, 1551 (C=C), 1231 (C–O). ^1^H NMR (DMSO-*d*_6_) *δ* 1.27 (s, 9H, *t*-Bu), 1.40 (s, 9H, *t*-Bu), 2.38 (s, 3H, *p*-CH_3_), 7.20 (d, 1H, *J* 2.4), 7.30 (d, 1H, *J* 2.2), 7.35 (d, 2H, *J* 8.1), 7.84 (d, 2H, *J* 8.1), 8.56 (s, 1H, CH=N), 12.14 (brs, 1H, NH), 12.29 (brs, 1H, OH). ^13^C NMR (DMSO-*d*_6_) *δ* 21.61 (C, p-CH_3_), 29.82 (3C), 31.83 (3C), 34.42 (C), 35.19 (C), 117 (C), 126.04 (C), 126.24 (C), 128.20 (2C), 129.67 (2C), 130.26 (C), 136.17 (C), 140.29 (C), 142.75 (C), 151.54 (C, C7, C=N), 155.54 (C), 163.16 (C, C=O). HREIMS found 366.2310 [M˙^+^] (calc. for C_23_H_30_N_2_O_2_, 366.2307).

#### *N*′-[(3,5-Di-*tert*-butyl-2-hydroxyphenyl) methylidene]-4-methoxybenzohydrazide (5b)

The crude product was recrystallised from ethanol to afford a white precipitate. Yield 1.08 g (95%); mp 258–260 °C; *ν*_max_ (KBr)/cm^−1^ 3662 (OH_phenol_), 3174 (NH), 3098 (CH_aromatic_), 3958 (CH_aliphatic_), 1662 (C=O), 1619 (C=N), 1596, 1572 (C=C), 1237 (C–O), 1095 (Ar–O–CH_3_). ^1^H NMR (DMSO-*d*_6_) *δ* 1.24 (s, 9H, *t*-Bu), 1.37 (s, 9H, *t*-Bu), 3.80 (s, 3H, OCH_3_), 7.05 (d, 2H, *J* 9.1), 7.16 (d, 1H, *J* 2.28), 7.26 (d, 1H, *J* 2.28), 7.89 (d, 2H, *J* 8.72), 8.51 (s, 1H, CH=N), 12.05 (brs, 1H, NH), 12.28 (brs, 1H, OH). ^13^C NMR (DMSO-*d*_6_) *δ* 29.30 (3C), 31.31 (3C), 33.88 (C), 34.67 (C), 55.52 (C, OCH_3_), 114.00 (2C), 117.18 (C), 124.73 (C), 125.56 (C), 125.77 (C), 129.73 (2C), 135.78 (C), 140.45 (C), 150.83 (C), 154.0 (C), 162.41 (C), 162.74 & 162.80 (2C). HREIMS found 382.2256 [M^+^] (calc. for C_23_H_30_N_2_O_3_, 382.2256).

#### *N*′-[(3,5-Di-*tert*-butyl-2-hydroxyphenyl)methylidene]-4-bromo-benzohydrazide (5c)

The crude product was recrystallised from aqueous acetonitrile to afford a white amorphous solid. Yield 1.22 g (95%); mp 276–278 °C; *ν*_max_ (KBr)/cm^−1^: 3660 (OH_phenol_), 3167 (NH), 3089 (CH_aromatic_), 2959, 1871 (CH_aliphatic_), 1664 (C=O), 1621 (C=N), 1995, 1558 (C=C), 1236 (C–O); ^1^H NMR (DMSO-*d*_6_) *δ* 1.27 (s, 9H, *t*-Bu), 1.40 (s, 9H, *t*-Bu), 7.22 (d, 1H, *J* 2.2), 7.31 (d, 1H, *J* 2.2), 7.78 (d, 2H, *J* 8.5), 7.88 (d, 2H, *J* 8.5), 8.56 (s 1H, C=N), 12.23 (1H, brs, NH), 12.28 (1H, brs, OH). ^13^C NMR (CDCl_3_) *δ* 29.81 (3C), 31.82 (3C), 34.44 (C), 35.18 (C), 117.38 (C), 125.38 (C), 126.25 (C), 126.44 (C), 130.22 (2C), 132.13 (3C), 136.01 (C), 140.91 (C), 152.06 (C), 155.04 (C), 162.30 (C). HREIMS found 430.1084 [M˙^+^] (calc. for C_22_H_27_BrN_2_O_3_, 430.1256).

#### *N*′-[(3,5-Di-*tert*-butyl-2-hydroxyphenyl) methylidene] 2-chloro-benzohydrazide (5d)

Recrystallisation of the crude product from ethanol afforded a white precipitate. Yield 1.03 g (90%); mp 138–140 °C; *ν*_max_ (KBr)/cm^−1^: 3675 (OH_phenol_), 3161 (NH), 3092 (CH_aromatic_), 2955, 2868 (CH_aliphatic_), 1651 (C=O), 1611 (C=N), 1231 (C–O); . ^1^H NMR (DMSO-*d*_6_) *δ* 1.28 (s, 9H, *t*-Bu), 1.42 (s, 9H, *t*-Bu), 7.23 (d, 1H, *J* 2.38), 7.32 (d, 1H, *J* 2.26), 7.46–7.63 (4H, m), 8.42 (1H, s, CH=N), 10.15 (1H, brs, NH), 12.23 (1H, brs, OH). ^13^C NMR (DMSO-*d*_6_) *δ* 29.26 (3C), 31.26 (3C), 33.87 (C), 34.62 (C), 116.81 (C), 125.70 (C), 125.89 (C), 127.30 (C), 129.42 (1C, C10), 129.79 (1C, C13), 130.47 (1C, C9), 131.58 (C), 134.69 (C), 135.63 (C), 140.41 (C), 151.42 (C, CH=N), 154.78 (C), 162.38 (C, CO), HREIMS found 386.1763 [M˙^+^] (calc. for C_22_H_27_ClN_2_O_2_, 386.1761).

### General synthesis of 2,4-di-*tert*-butyl-6-[5-(substitutephenyl)-1,3,4-oxadiazol-2-yl]phenol (6a–d)

To a solution of *N*′-[(3,5-di-*tert*-butyl-2-hydroxyphenyl) methylidene]-substituted benzohydrazide (2 mmol) in 10 mL glacial acetic acid and anhydrous sodium acetate (2 mmol) in a 50 mL round-bottom flask, bromine (1 mmol in 3 mL ACOH) was added dropwise at ambient temperature with vigorous stirring. The mixture was stirred for 1 h and then refluxed further for 2 h. Upon cooling, the mixture was poured into 50 mL ice water. The resulting precipitate was collected and washed with distilled water, dried and either purified by column chorography or recrystallised with a suitable solvent.

#### 2,4-Di-*tert*-butyl-6-[5-(4-methylphenyl)-1,3,4-oxadiazol-2-yl]phenol (6a)

The crude material was purified by column chromatography using (hexane-ethyl acetate) 6–1 as elute to give a white crystal. Yield 0.50 g (70%) yield; mp 184–186 °C; *ν*_max_ (KBr)/cm^−1^: 3474 (br, OH_phenol_), 3082 (CH_aromatic_), 2962, 2855 (CH_aliphatic_), 1609 (C=N), 1590, 1552 (C=C), 1219 (C–O); ^1^H NMR (CDCl_3_) *δ* 1.40 (s, 9H, *t*-Bu), 1.44 (s, 9H, *t*-Bu), 2.43 (s, 3H, CH_3_), 7.33 (d, 2H, *J* 8.0), 7.49 (d, 1H, *J* 2.44), 7.67 (d, 2H, *J* 2.44), 8.02 (d, 2H, *J* 8.0), 10.57 (brs, 1H, OH). ^13^C NMR (CDCl_3_) *δ* 21.68 (C, p-CH_3_), 29.41 (3C), 31.47 (3C), 34.37 (C), 35.32 (C), 107.63 (C), 120.50 (C), 120.68 (C), 126.99 (2C), 128.37 (C3), 129.82 (2C), 137.45 (C), 141.60 (C) 142.58 (C), 154.68 (C), 163.16, 164.97 (C7 & C8). HREIMS found 364.2162 [M^+^] (calc. for C_23_H_28_N_2_O_2_, 364.2151).

#### 2,4-Di-*tert*-butyl-6-[5-(4-methoxyphenyl)-1,3,4-oxadiazol-2-yl]phenol (6b)

The crude product was recrystallised from methanol-chloroform to afford white crystals. Yield 0.56 g (74%); mp 170–172 °C; *ν*_max_ (KBr)/cm^−1^: 3470 (br, OH_phenol_), 3090 (CH_aromatic_), 2959, 2868 (CH_aliphatic_), 1610 (C=N), 1585, 1547 (C=C), 1222 (C–O), 1170 (O–CH_3_). ^1^H NMR (CDCl_3_) *δ* 1.37 (s, 9H, *t*-Bu), 1.48 (s, 9H, *t*-Bu), 3.87 (s, 3H, OCH_3_), 7.06 (d, 2H, *J* 8.8), 7.51 (d, 1H, *J* 2.2), 7.68 (d, 2H, *J* 2.4), 8.10 (d, 2H, *J* 8.8), 10.58 (brs, 1H, OH). ^13^C NMR (CDCl_3_) *δ* 29.42 (3C), 31.48 (3C), 34.38 (C), 35.33 (C), 3.51 (C, OCH_3_), 107.69 (C), 114.60 (2C), 115.96 (C), 120.46 (C), 128.30 (C), 128.87 (2C), 137.44 (C), 141.58 (C), 154.63 (C), 162.62 (C), 162.56, 164.78 (C7 & C8). HREIMS found 380.2102 [M˙^+^] (calc. for C_23_H_28_N_2_O_3_, 380.2100).

#### 2,4-Di-*tert*-butyl-6-[5-(4-bromophenyl)-1,3,4-oxadiazol-2-yl]phenol (6c)

The crude product was recrystallised from THF to afford a white precipitate. Yield 0.67 g (79%); mp 158–160 °C, *ν*_max_ (KBr)/cm^−1^: 3462 (b, OH_phenol_), 3088 (CH_aromatic_), 2955, 2842 (CH_aliphatic_), 1608 (C=N), 1587, 1552 (C=C), 1214 (C–O), ^1^H NMR (CDCl_3_) *δ* 1.37 (s, 9H, *t*-Bu), 1.48 (s, 9H, *t*-Bu), 7.53 (d, 1H, *J* 2.2), 7.67 (d, 1H, *J* 2.4), 7.70 (d, 2H, *J* 8.5), 8.03 (d, 2H, *J* 8.5), 10.51 (brs, 1H, OH); ^13^C NMR (CDCl_3_) *δ* 29.42 (3C), 31.47 (3C), 34.41 (C), 35.36 (C), 113.50 (C), 120.51 (C), 122.41 (C), 126.73 (C), 128.45 (2C), 128.76 (C), 132.64 (2C), 137.64 (C), 141.77 (C), 154.84 (C), 162.94 & 165.42 (C7 & C8), HREIMS, found 428.1102 [M˙^+^] (calc. for C_22_H_25_BrN_2_O_2_, 428.1099).

#### 2,4-Di-*tert*-butyl-6-[5-(2-chlorophenyl)-1,3,4-oxadiazol-2-yl]phenol (6d)

The crude product was recrystallised from ethanol-chloroform to yield white needle-like crystals. Yield 0.61 g (80%) yield; mp 138–140 °C; *ν*_max_ (KBr)/cm^−1^: 3475 (br, OH_phenol_), 3094 (CH_aromatic_), 2968, 2857 (CH_aliphatic_), 1612 (C=N), 1585, 1547 (C=C), 1221 (C–O), ^1^H NMR (CDCl_3_) *δ* 1.34 (s, 9H, *t*-Bu), 1.48 (s, 9H, *t*-Bu), 7.70–7.44 (m, 4H), 7.69 (d, 1H, *J* 2.4), 8.07 (dd, 1H, *J* 7.56, 1.95), 10.46 (brs, 1H, OH). ^13^C NMR (CDCl_3_) *δ* 29.46 (3C), 31.47 (3C), 34.41 (C), 35.39 (C), 107.42 (C), 120.86 (C), 122.82 (C), 127.24 (C), 128.7 (C), 131.27 (C, C10), 131.46 (C), 132.67 (C), 133.39 (C), 137.60 (C), 141.83 (C), 154.84 (C), 161.43 (C), 165.66 (C). HREIMS found 384.1605 [M˙^+^] (calc. for C_22_H_25_ClN_2_O_2_, 384.1605).

### DPPH radical scavenging activity

The DPPH free radical scavenging assay was carried out using Brand–Williams' method^37,38^ with slight adjustments. 195 μL of a 100 μM methanolic DPPH solution was mixed with 50 μL of the test compounds at various doses (0–1000 μg mL^−1^) to make the reaction mixture. Initially, the test chemicals were dissolved in dimethyl sulphoxide (DMSO). Positive controls were BHT and ascorbic acid, which were tested in tandem. The absorbance of the reaction mixture was measured at 570 nm after 60 minutes of incubation in the dark at room temperature. As a result of the lower absorbance, the colour of the reaction mixture changed from purple to yellow. The following equation was used to compute the radical scavenging activity:1
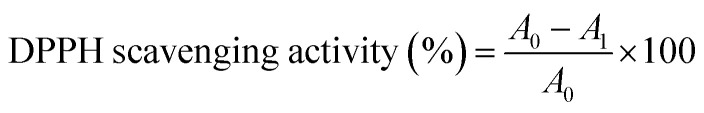
where *A*_0_ is the absorbance of the DPPH radical without a sample or standard; and *A*_1_ is the absorbance of the DPPH radical with a sample or standard. The IC_50_ values were determined and given in μg mL^−1^, which represent the effective concentration of the standards and samples that inhibit 50% of the DPPH radicals.^39–41^

### Ferric reducing antioxidant power activity (FRAP)

FRAP was determined according to the method described by Benzie and Strain^38,42^ with slight modifications. Three reagents were initially prepared as follows: 300 mM acetate buffer (pH = 3.6), 10 mM 2,4,6-tripyridyl-*s*-triazine (TPTZ) in 40 mM HCl and 20 mM FeCl_3_.

The acetate buffer was mixed with the TPTZ solution in 20 mM FeCl_3_ at a ratio of 10 : 1 : 1 (v/v/v) to prepare a fresh FRAP working solution. Five microliters of the samples or standards were mixed with 300 mL of the FRAP reagent and then incubated at 37 °C for 30 minutes. The absorbance of the stained product was then measured at 595 nm. Based on a calibration curve prepared with ferrous sulfate (FeSO), the results were determined (0–1 mM). The results were expressed in FRAP value.

### Statistical analysis

All of the analyses were carried out in triplicate. The data was presented as a mean with a standard deviation. The association between the antioxidant activity of the synthesised compounds in the DPPH and FRAP antioxidant assays was investigated using a correlation coefficient test. At the 0.05 level, the trend is significant. OriginPro software, version 2016, was used to statistically analyse the data.

### Computational details

The Gaussian 09 programme package was used to accomplish all of the computations provided here.^43^ All naturally occurring systems, including neutrals, radicals, radical cations, and anions, were subjected to geometry optimisation and frequency analysis in vacuum. At the B3LYP/6-311++g(d,p) level of theory,^44,45^ they were entirely optimised in their electronic ground states, as evidenced by the absence of imaginary frequencies. Open-shell systems lacking spin impurities, such as radicals and radical cations, were subjected to unconstrained calculations. In all situations, the expectation value of 〈*S*_2_〉 operator was around 0.750. At the same level of this structural optimisation, the HOMO and LUMO frontier molecular orbitals were depicted for neutral compounds, and global reactivity descriptors were derived.

## Results and discussion

### Chemistry

All compounds were characterised by IR, ^1^H NMR, ^13^C NMR, EIMs and HREIMs. HSQC was used for 4c to distinguish between the SCH_2_ carbon from the three similar peaks at 34.38, 35.32 and 36.16 ppm. HMBC was used for 5b to distinguish between OH and NH protons in hydrazones. HMBC was also used for 6a to distinguish between the carbons of the oxadiazole ring.

#### Characterisation of synthesised compounds

2,4-Di-*tert*-butyl benzoic acid was first converted to its methyl ester and then to the hydrazide. The hydrazide was then reacted with carbon disulphide in the presence of potassium hydroxide to yield compound 3. The IR spectrum showed the disappearance of the NH_2_ and the carbonyl bands. New bands appeared at 1618 cm^−1^ for C=N and 1246 cm^−1^ for C=S. From NMR spectroscopic data, all protons were accounted for compounds 4a–c were synthesised by the alkylation of compound 3 which were selectively reacted at the thione group (without affecting the phenol group) at ambient temperature. The HSQC of compound 4c displayed the correlation between the protons of the SCH_2_ group and their carbons (C–H) at 4.4–36.16 ppm; therefore, the other two peaks must belong to the quaternary carbons at the di-*tert*-butyl group. The hydrazones 5a–d were successfully synthesised in high yield. The HMBC of compound 5b was used to distinguish between the protons of OH and NH through long-distance coupling for *J*_3_ and weak coupling for *J*_2_. The most important correlations were those between the hydrogen of phenol hydroxyl at 12.27 ppm with C6, C2 and C1, whereas the NH at 12.04 ppm showed correlation with carbonyl group C8. The proton of the imine group (CH=N) showed a correlation with C1 and C6. In addition, the C1 displayed a long-range correlation with C7, C3 and C5. The protons of the 4-bromo phenyl ring also displayed their own correlation, whereas the H10 exhibited a correlation with C12 and C8 even though H11 showed a correlation with H9. The cyclisation of hydrazones was carried out in the presence of bromine as an oxidising agent. The formation of oxadiazoles 6a–d was confirmed by all spectroscopic data available. Furthermore, HMBC was used to distinguish between the two carbons of the oxadiazole ring, C7 and C8. Compound 6a was used as an example to illustrate our results. The correlation between H10 and C12 and C8 as well as the correlation between H5 and C3, C1 and C7 can be clearly observed in the HMBC spectrum. These data help us to distinguish between the two carbons in the oxadiazole ring. Detailed information can be accessed in the ESI† for the respective HSQC (Fig. 9†) and HMBC (Fig. 21†). The structure of compounds 6a–d were further confirmed by the fragmentation pattern shown on the EIM spectra, which revealed the loss of isocyanic acid (see Section 1.2 of the ESI†).

### Evaluation of antioxidant assay

#### DPPH assay

**Fig. 3:**
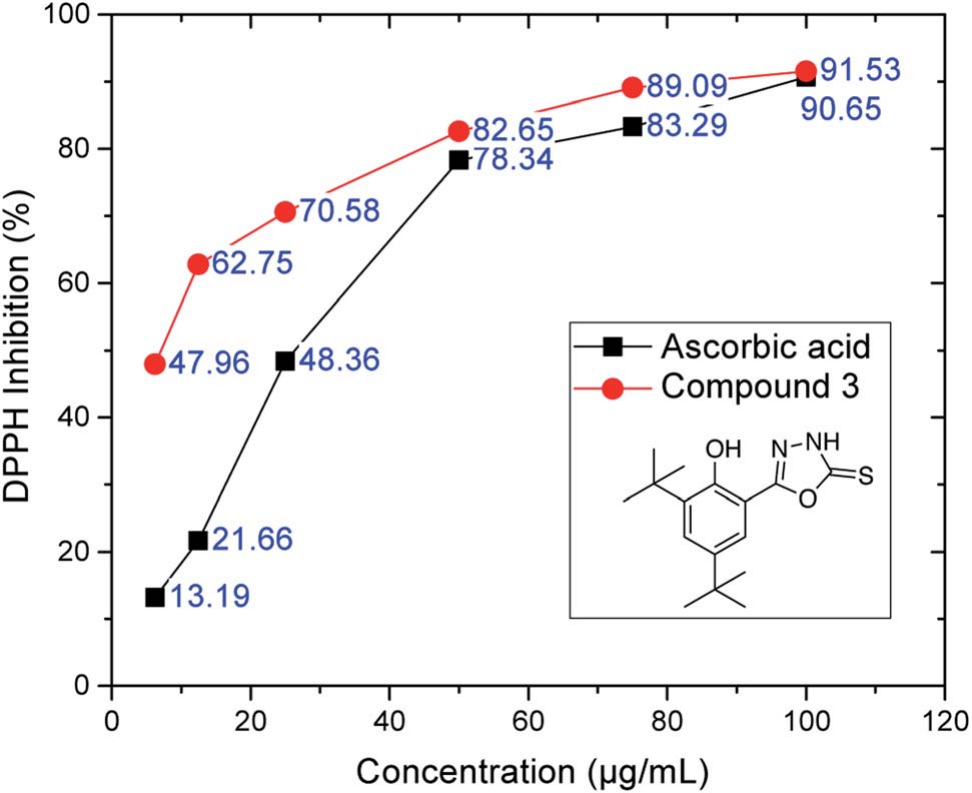
DPPH inhibition of oxadiazole-thione 3 at six different concentrations. Ascorbic acid as a reference compound.

The antioxidant capacity of the compounds was studied by considering their scavenging activity against DPPH radicals.^46^ The percentage of DPPH inhibition and the IC_50_ values (half maximal scavenging concentration) were calculated with reference to the DPPH absorbance (0%). The oxadiazole-thione derivative (compound 3) showed extraordinary antioxidant ability in DPPH compared to the other compounds. High antioxidant activity was also observed in DPPH inhibition at low concentrations, *e.g.*, 12.5 μg mL^−1^ showed an inhibition value of 62.75%, whereas ascorbic acid showed an inhibition value of 21.66% (Fig. 3).

However, the thio-oxadiazole compounds (4a–c) exhibited lower antioxidant activity than compound 3, which led us to conclude that the oxadiazole-thione groups behave as better free radical scavengers.^47^ The NHCS group can be regarded as a part of the thiourea system, which is known as an effective antioxidant.^48^ The similarity of this case with that of phenethyl-5-bromo-pyridyl thiourea^49^ further supports our conclusion. In the series of thio-oxadiazole, compound 4c (IC_50_: 43.62 ± 0.12 μg mL^−1^), with 4-bromo benzyl group next to the thio group afforded better antioxidant activity when compared to the activities observed with methyl (4a, IC_50_: >100 μg mL^−1^) or propargyl groups (4c, IC_50_: 43.62 ± 0.12 μg mL^−1^). In the next series, only hydrazone 5a recorded a good IC_50_ value (73.77 ± 0.11 μg mL^−1^), which is comparable to BHT. In the series of phenyloxadiazoles, two compounds (6a, IC_50_: 72.47 ± 0.28 μg mL; 6d, IC_50_: 77.42 ± 0.80 μg mL^−1^), recorded comparable IC_50_ values to that of BHT (IC_50_: 79.84 ± 0.15 μg mL^−1^). Their phenyloxadiazoles (6a–d) exhibited slightly lower antioxidant activity than that of the hydrazones. In this case, different functional groups and heterocyclic rings clearly plays an important role in increasing and decreasing the antioxidant activity, as depicted in Table 1.

**Table 1:** The antioxidant properties of synthesised compounds

Compound	R	Inhibition ± SD^a^ 100 μg mL^−1^	IC_50_ ± SEM^b^ μg mL^−1^	FRAP value^c^ μM
3	—	91.53 ± 0.11	6.13 ± 0.63	4612.78
4a	CH_3_	40.85 ± 0.17	>100	297.78
4b	CH_2_C≡CH	37.63 ± 0.64	>100	291.94
4c	CH_2_Ph–4Br	62.59 ± 0.92	43.62 ± 0.12	1016.16
5a	4-CH_3_	61.98 ± 0.61	73.77 ± 0.11	538.67
5b	4-OCH_3_	51.48 ± 0.29	>100	511.17
5c	4-Br	36.11 ± 0.16	>100	278.67
5d	2-Cl	48.36 ± 0.13	>100	433.67
6a	4-CH_3_	61.79 ± 0.37	72.47 ± 0.28	520.33
6b	4-OCH_3_	44.76 ± 0.19	>100	359.50
6c	4-Br	26.46 ± 0.44	>100	112.83
6d	2-Cl	54.68 ± 0.50	77.42 ± 0.80	468.67
BHT	—	66.03 ± 0.22	79.84 ± 0.15	488.30
Ascorbic acid	—	90.65 ± 0.27	22.71 ± 0.23	848.90
Quercetin	—	—	—	2090.60
Gallic acid	—	—	—	2421.10

a Standard deviation (SD) value in FRAP was between 0.01–0.16.

b SEM Standard error of the mean and IC_50_: 50% of effective concentration.

c Dosage: 100 μg mL^−1^.

Looking at the percentage of DPPH inhibition, the effect of the substituted group in compounds 5a–d ranked as follows: 4-methyl > 4-methoxy > 2-chloro > 4-bromo; on the other hand, compounds 6a–d showed the following sequence: 4-methyl > 2-chloro > 4-methoxy > 4-bromo. Compounds 6a–d share the same basic structures; the only difference is the position of the substituted group at the phenyl ring. This finding led us to conclude that the nature and position of each substituted group play some role in enhancing or inhibiting the antioxidant ability of the hydrazones and phenyloxadiazoles.

#### FRAP assay

The FRAP assay determined the antioxidant activity of the compounds by measuring their reducing potentials. The reducing potential of the compounds is measured based on their ability to reduce the colorless [Fe^3+^-(2,4,6-tris(2-pirydyl)-*s*-triazine)_2_]^3+^ complex to the intensive blue-colored complex [Fe^2+^-(TPTZ)_2_]^2+^ in acidic medium, which is quantified by measuring the color changes spectrophotometrically at 593 nm, where the increase in absorbance is proportional to the reducing potential of the compounds.^50,51^ Obtained results are calculated from a standard curve constructed using a standard Fe^2+^ ion solution. The results are expressed in FRAP units, where one FRAP unit can be defined as the reduction of 1 M Fe^3+^ ion to one Fe^2+^ ion.^51^ The acidic medium (pH 3.6) is required to facilitate Fe^3+^ complex solubility, yet the condition also results in lower ionisation potential, which promotes the single electron transfer mechanism, as opposed to the hydrogen atom transfer mechanism in DPPH assay (Scheme 38 in ESI†).^52^

**Fig. 4:**
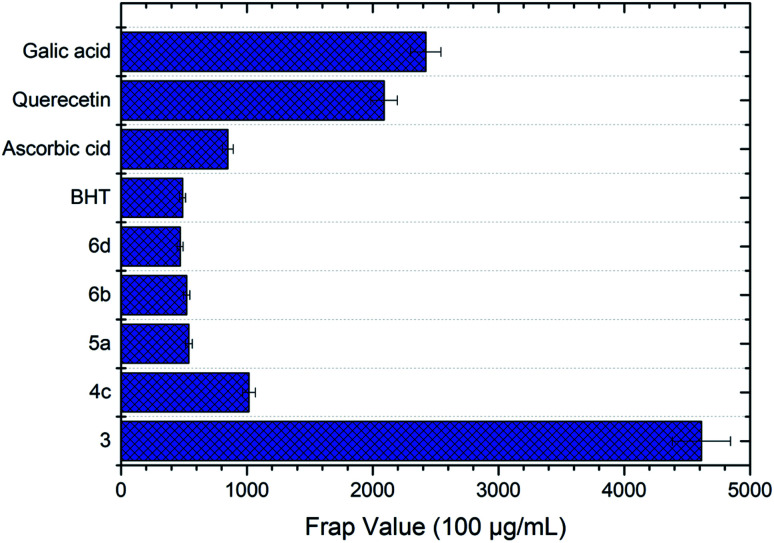
FRAP value of the oxadiazole-thione 3, the thio-oxadiazole series (4c), the hydrazone series (5a) and the phenyloxadiazole series (6b & 6d) at concentrations of 100 μg mL^−1^. BHT, ascorbic acid, quercetin and gallic acid as reference compounds.

**Scheme 1:**
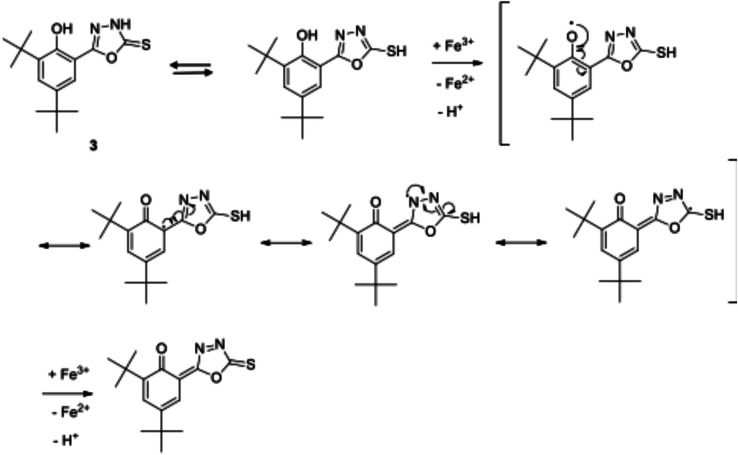
Resonance structures of the radical form of oxadiazole-thione 3.

Compound 3 exhibited the best antioxidant activity among other compounds with the FRAP value of 4612.78 μM, which was also significantly higher as compared to the reference compounds gallic acid (2421.10 μM), quercetin (2090.60 μM), BHT (488.3 μM) and ascorbic acid (848.9 μM) (see Fig. 4). The high potency activity of compound 3 can be explained by the stability of the resonance of its radical structures (Scheme 32†). The activation of the hydroxyl group, by electron transfer followed by removal of proton, leads to the activation of the thiol group.^52^ This reaction allows one mole of oxadiazole-thione 3 to reduce two moles of Fe^3+^ ion, and consequently, give a high FRAP value and potent antioxidant property for oxadiazole-thione 3 (Scheme 1).

In the series of thio-oxadiazole compounds, compound 4c, with 4-bromobenzyl substituent, recorded the highest FRAP value at 1016.16 μM, significantly higher than compound 4a (297.78 μM) and 4b (291.94 μM), which were consistent to DPPH results. Similarly, as in DPPH results, compound 5a showed the highest antioxidant property in FRAP assay (538.67 μM) as compared to compound 5b (511.17 μM), 5c (278.67 μM) and 5d (433.67 μM). In the series of phenyloxadiazole compounds, compound 6a, with 4-methyl substituent, displayed the best antioxidant activity with the FRAP value of 520.33 μM, as compared to compound 6b (359.50 μM), 6c (112.82 μM), and 6d (468.67 μM). The results of FRAP assay further reinforced that oxadiazole-thiones possessed higher antioxidant properties as compared to thio-oxadiazoles, hydrazones and phenyloxadiazoles, which were previously exhibited in DPPH assay.

#### Pearson correlation analysis

**Fig. 5:**
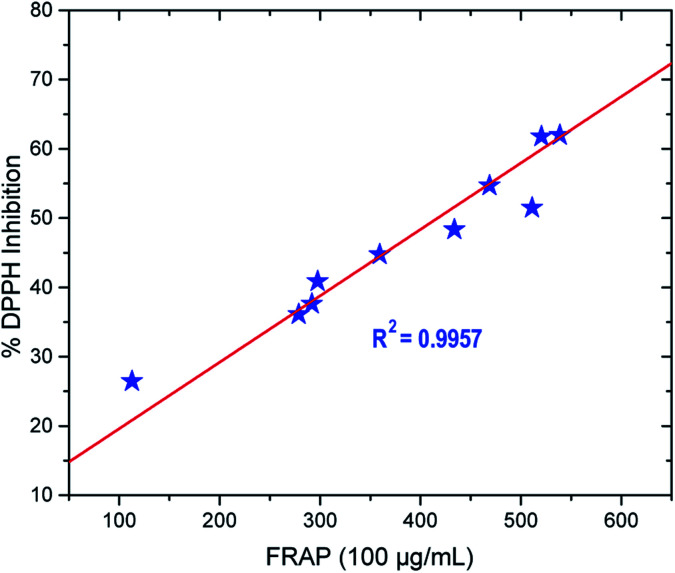
Pearson correlation analysis between DPPH radical scavenging and iron (iii) reducing activities of the synthesised compounds for the series of thio-oxadiazole (4a–c), hydrazones (5a–d) and phenyloxadiazole (6a–d). The correlation is significant at the 0.05 level.

A Pearson correlation analysis was performed to explore the relationship between the DPPH radical scavenging and iron (iii)-reducing activities of the compounds,^38^ as depicted in Fig. 5. It was expected that the antioxidant activities of the synthesised compounds for the series of thio-oxadiazole (4a–c), hydrazones (5a–d) and phenyloxadiazole (6a–d) in both assays would show similar trends and that the values of two different mechanisms could therefore be correlated. The results revealed a strong positive correlation between the two antioxidant assays, demonstrating that compounds with high DPPH radical scavenging activity also have significant iron reduction activity.

### Density functional theory

#### Frontier molecular orbital

**Fig. 6:**
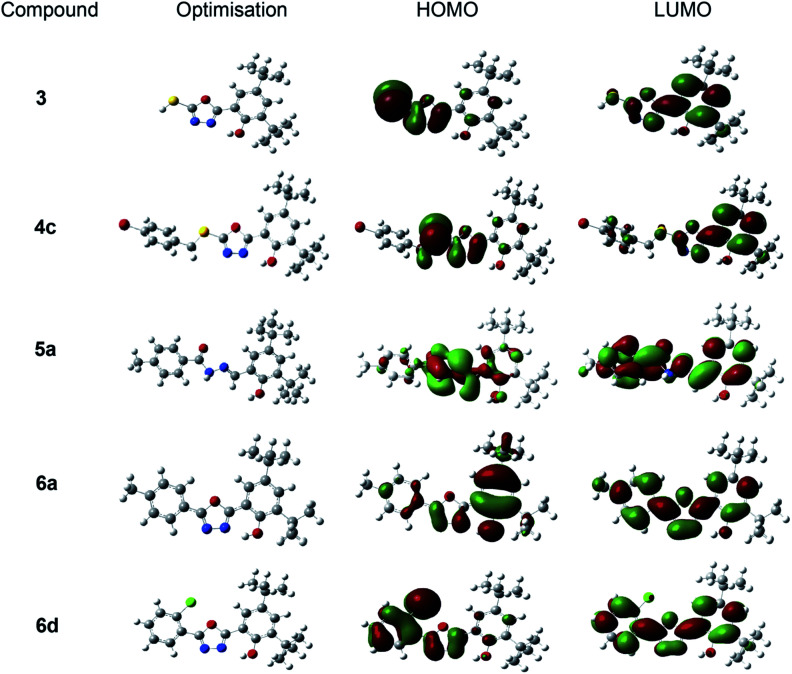
Depictions of the optimised conformations of the most stable structures and frontier molecular orbital plots (HOMO and LUMO) of oxadiazole-thione 3, thio-oxadiazole 4c, hydrazone 5a and phenyloxadiazole 6a & 6d in the gas phase at B3lyp/6-311++g(d,p).

The ability to describe the scavenging behaviour of antioxidant compounds requires a thorough understanding of their electrical and structural features. As a result, the structures of compounds that displayed good activities in DPPH and FRAP assays were chosen to undergo comprehensive optimisation. The MM + force field,^53^ which was implemented in the HyperChem program,^54^ was utilised to perform molecular mechanics optimisation of the conformation search, the results were used for further topology optimisation at the B3LYP/6-311++g(d,p) level of theory. Fig. 6 shows the most stable geometries that were selected for the preparation of input geometries of radicals for optimisation and frontier molecular orbital investigations.

Fig. 6 depicts the gas-phase electron density distribution of the highest occupied molecular orbital (HOMO) and the lowest unoccupied molecular orbital (LUMO) for oxadiazole-thione 3, thio-oxadiazole (4c), hydrazone 5a and the phenyloxadiazole (6a & 6d), which were calculated based on their good antioxidant activity. The HOMO plots demonstrate that HOMOs are localised on the oxadiazole ring and hydrazones bonds in the investigated systems. As a result, the oxadiazole ring and hydrazone would be the most likely reaction site for free radicals to assault and remove an electron. The distribution of LUMOs, on the other hand, demonstrates that the entire molecules are heavily contributed. Compound with smaller molecular orbital gap at the Frontier is more polarised,^58^ thus, it is expected to have higher intermolecular charge transfer between election donors and acceptors, which increases/decreases its biological activity.^59^

Table 2 shows that the oxadiazole-thione, 3 has a smaller HOMO–LUMO gap (0.085 a.u) compared to a series of phenyloxadiazolephenyloxadiazole; phenyloxadiazole 6a (0.173 a.u) and 6d (0.170 a.u), followed by hydrazone thio-oxadiazole 4c (0.804 a.u) and 5a (0.182 a.u.), indicating that it has high polarizability. The polarizability of molecules is used to determine the relative tendency of charge distribution. In other words, in the presence of a weak external electric field, the system electronic cloud can be deformed from its typical shape.^60^

**Table 2:** Selected Reactivity Descriptors such as *ε*_HOMO_, *ε*_LUMO_, Δ*E*_HOMO–LUMO_, dipole moment and O–H BDE, at the B3LYP/6-311++g(d,p) level of theory in the gas phase

Molecule	*ε*_HOMO_ a	*ε*_LUMO_ a	Δ*E*_HOMO–LUMO_^a^	Dipole moment^a^	BDE^b^	IP^b^
3	−0.233	−0.148	0.085	3.615	50.05	145.88
4c	−0.244	−0.064	0.180	2.216	55.65	153.01
5a	−0.241	−0.059	0.182	4.621	54.85	155.16
6a	−0.242	−0.069	0.173	4.678	59.98	155.38
6d	−0.247	−0.077	0.170	3.774	59.55	156.72
BHT^55^	—	—	—	—	70.14	171.90
Ascorbic acid^55^	—	—	—	—	74.54	196.82
Quercetin^56^	—	—	—	—	65.80	150.32
Gallic acid^57^	—	—	—	—	77.10	146.70

a All parameters in atomic unit.

b All values in kcal mol^−1^.

#### Descriptors of the antioxidant properties

The properties of antioxidant activity can be described several parameters, including bond dissociation energy (BDE) and ionisation potential (IP) (Table 2). In the context of HAT mechanism, where a hydrogen atom is transferred from a hydroxyl group of an antioxidant to a free radical, the most relevant parameter is the BDE.^61^ It is expected that O–H bond has the lowest BDE is the weakest, and its hydrogen is the easiest to be abstracted, revealing compound with highest antiradical (antioxidant) activity.

Table 2 shows the calculated BDE values of the OH bond for the compounds in the gas phase. It is shown that oxadiazole-thione 3 has the lowest BDE value (50.05 kcal mol^−1^), indicating the highest radical scavenging activity as compared to the other compounds, which is consistent with the experimental results of the DPPH assay.

The pattern of BDE values of thio-oxadiazole 4c (55.65 kcal mol^−1^) and phenyloxadiazoles 6a & 6d (59.98, 59.55 kcal mol^−1^) is consistent with the result of DPPH assay, in which thio-oxadiazole 4c displayed higher antiradical activity as compared to phenyloxadiazoles 6a and 6b. Meanwhile, the similarity of the BDE values of phenyloxadiazoles 6a and 6d are also reflected in their IC_50_ values in DPPH assay (72.47 μg mL^−1^, 77.42 μg mL^−1^). Only the BDE value of hydrazone 5a (54.85 kcal mol^−1^) which is slightly lower than thio-oxadiazole 4c, could not explained its activity in DPPH assay (73.77 μg mL^−1^), which is higher than thio-oxadiazole 4c.

In comparison to the BDE value of BHT as the reference (70.14 kcal mol^−1^), oxadiazole-thione 3 and thio-oxadiazole 4c recorded lower BDE values, which are in agreement to the results of DPPH assay. From the BDE values, it can be deduced that the oxadiazole-thione group at the *ortho* position promotes the HAT mechanism of the butylated phenol group, and this is followed by the hydrazones, thio-oxadiazoles, and phenyloxadiazoles groups at lower extent, respectively.

Other than BDE, another parameter that can measure antioxidant property is ionisation potential (IP) or proton dissociation enthalpy (PDE), which describes the energetics of the SET-PT process.^62^ Compound with lower IP value has higher likelihood to produce superoxide anion radical, by immediately passing the electron to the environment.^63^ The calculated IP of the compounds are recorded in Table 2.

The obtained results reveal that the IP values of the five selected synthesised compounds are almost equivalent. Oxadiazole-thione 3 has the lowest IP value (145.88 kcal mol^−1^), as compared to the rest of the synthesised and reference compounds, and this is reflected in the results of FRAP assay. Meanwhile, thio-oxadiazole 4c, which recorded higher IP value (153.01 kcal mol^−1^) than quercetin (150.32 kcal mol^−1^) and gallic acid (146.70 kcal mol^−1^), but lower than compounds 5a (155.16 kcal mol^−1^), 6a (155.38 kcal mol^−1^) and 6d (156.72 kcal mol^−1^), has a consistent pattern in the FRAP assay, in which it recorded lower activity that quercetin and gallic acid, but higher than compounds 5a, 6a and 6d. Similar to BDE parameter, the IP values showed that the oxadiazole-thione group at the *ortho* position promotes the SET-PT mechanism of the butylated phenol group, followed by the thio-oxadiazoles, hydrazones and phenyloxadiazoles groups at lower extent, respectively.

## Conclusions

12 derivatives of butylated phenol with four different groups at the *ortho* position were synthesised and studied for their antioxidant properties. Oxadiazole-thione 3, which features oxadiazole-thione ring at the *ortho* position, recorded the best result in both DPPH (IC_50_: 6.13 μg mL^−1^) and FRAP (FRAP value: 4612.78) assays, which were significantly better than the reference compounds, including BHT, ascorbic acid, quercetin and gallic acid. The potent antioxidant activity of oxadiazole-thione 3 was explained by the stability of the resonance structure of its radical form, as well as the calculated parameters BDE and IP. Based on the experimental and calculated results, the order of groups at the *ortho* position of butylated phenol that contribute to higher antioxidant activity can be written as follow: oxadiazole-thione > thio-oxadiazole > hydrazone > phenyloxadiazole. Oxadiazole-thione 3 is a good candidate to be further investigated and developed as an antioxidant.

## Author contributions

The authors report the following individual contributions to this work: formal analysis: RS, NN, AA; funding acquisition and project administration: NN, AA; investigation: RS, NN; resources: NN, AA; software: NN; supervision: AA, NN; conceptualisation, data curation, methodology, validation, writing – original draft, writing – review and editing: all.

## Conflicts of interest

There are no conflicts to declare.

## Supplementary Material

RA-012-D2RA02140D-s001
