# Supplementary material for: Antioxidant properties of butylated phenol with oxadiazole and hydrazone moiety at ortho position supported by DFT study
Source: RSC Adv. 2022 Jun 9;12(27):17085–95. doi: 10.1039/d2ra02140d (PMC9178441; doi:10.1039/d2ra02140d)
Supplement: RA-012-D2RA02140D-s001 [file RA-012-D2RA02140D-s001.pdf]

Electronic Supplementary Material (ESI) for RSC Advances.
This journal is © The Royal Society of Chemistry 2022

Supplementary Information

Antioxidant Properties of Butylated Phenol with Oxadiazole and Hydrazone moiety at *ortho* position supported by DFT Study

Raied M. Shakir, Muhammad Kumayl Abdulwahab, Nurdiana Nordin*, and Azhar Ariffin*

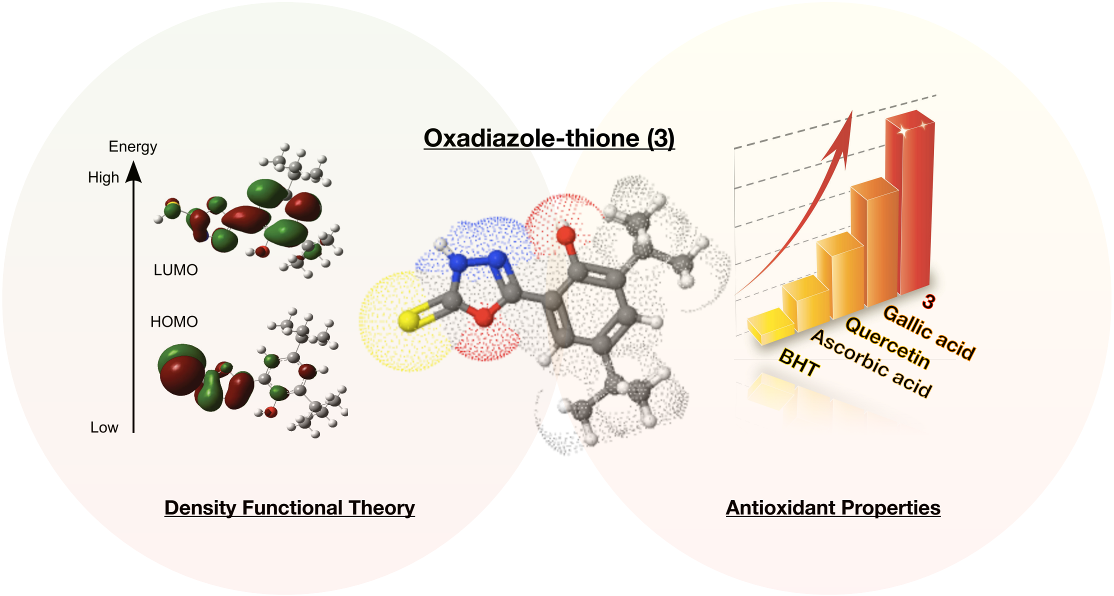

Two series of 1,3,4-oxadiazole derivatives at the sixth position of the 2,4-di-*tert*-butylphenol group were synthesized. The antioxidant properties were evaluated by DPPH and FRAP assays. Compound 3 showed significant antioxidant activity, while its alkyl derivatives exhibited decreased antioxidant activity in both assays. The preferential antioxidant mechanism of the reactive antioxidant molecules prepared from the further reaction of compound 3 to produce compounds 4 and 6 was investigated using density functional theory. Calculating their comprehensive reactivity descriptors was used to assess their antioxidant reactivity. According to the calculated descriptors, compounds 4c and 6d are the most reactive antioxidants within their own group compared to the other derivative moieties. The results are identical to ascorbic acid's, indicating that they have similar activity. The experimental data and the calculated descriptors are in good agreement. The nature of the substituents and their positions have a significant impact on the derivatives' antioxidant capabilities.

Keywords— di-*tert*-butyl phenol (DTBP), antioxidant, 1,3,4-oxadiazole, hydrazones, losing HNCO, DFT, BDE, IP

Contents

1 Supporting Information 1: Chemistry **2**

1.1 Spectra 2

1.2 The Mass and Loss of HNCO 19

2 Supporting Information 2: Theoretical and Computational details **20**

2.1 Optimisation of the Range-Separation Parameter. 20

2.2 Radical Scavenging Pathways 20

2.3 Proposed resonance structures of FRAP mechanism 21

1 Supporting Information 1: Chemistry

1.1 Spectra

As characterisation experiments, a one-dimensional (1D) NMR experiments were performed on the synthesised compounds in solutions at ≈ 25°C to identify the relevant signals. All chemical shifts are expressed in units of parts per million (ppm) with respect to TMS (0≈ppm).

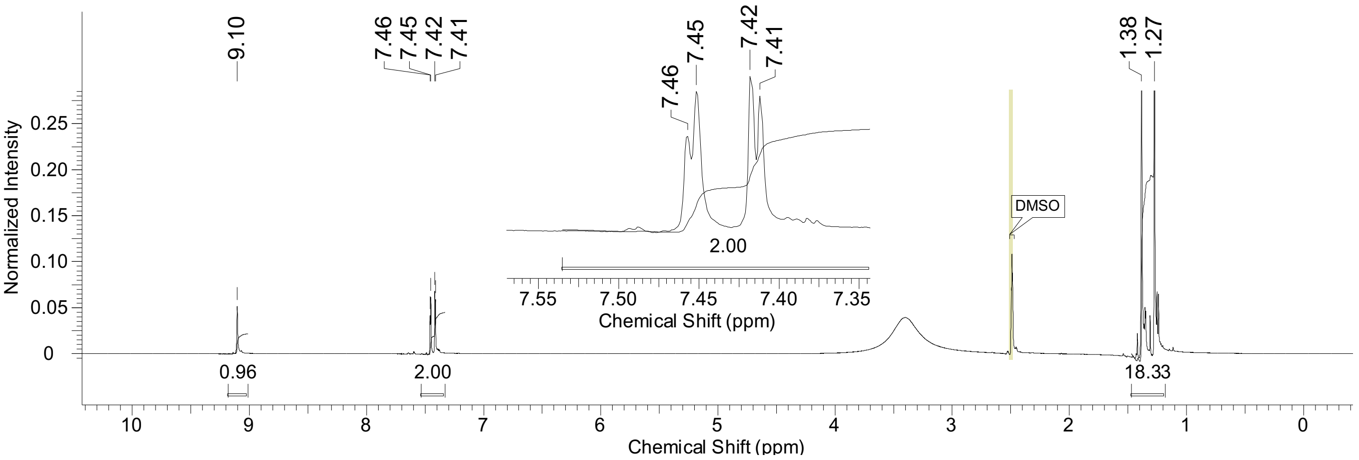

Figure 1 ^{1}H NMR of **3**

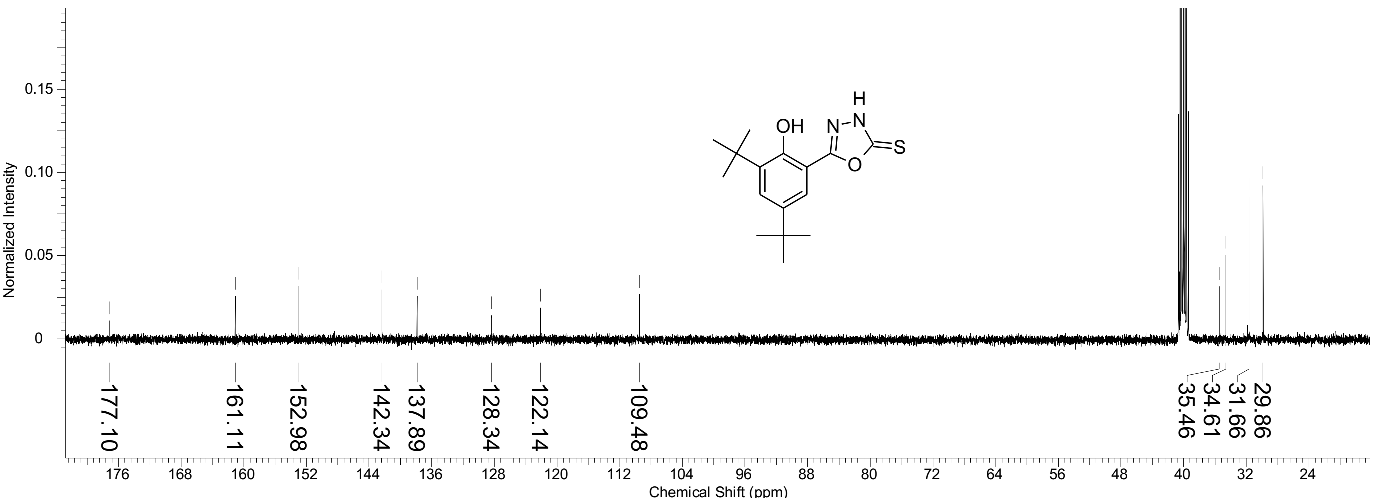

Figure 2 ^{13}C NMR of **3**

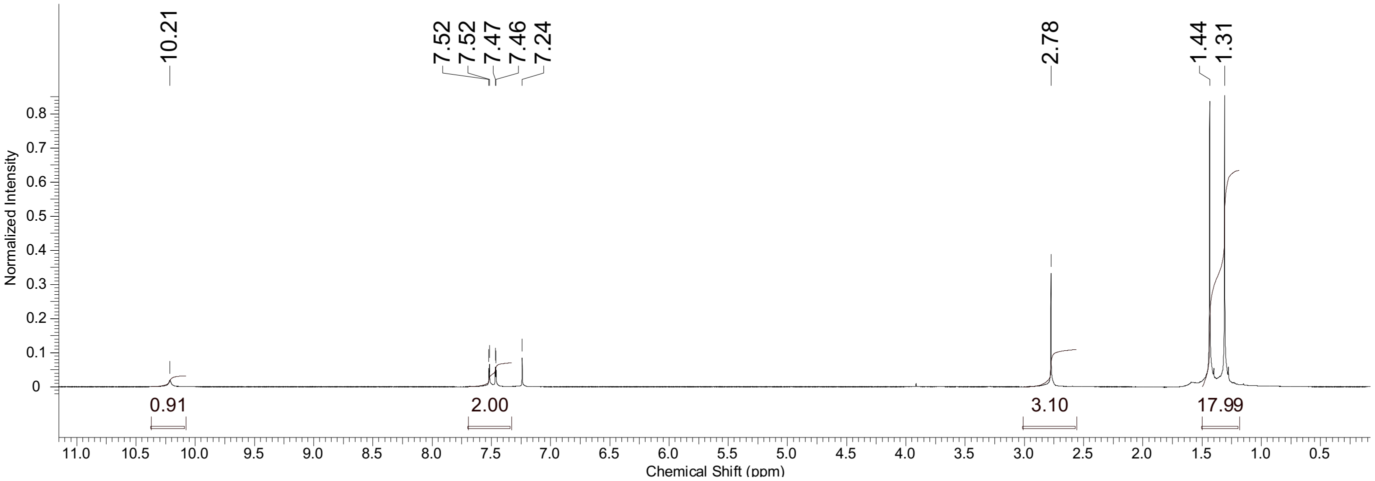

Figure 3 ^{1}H NMR of **4a**

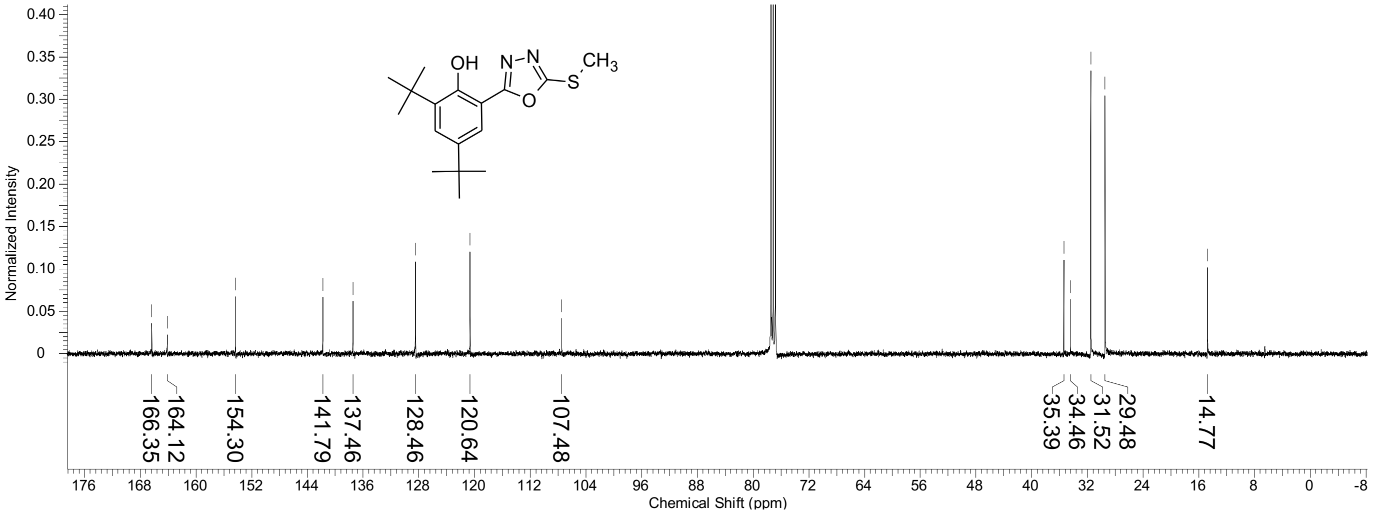

Figure 4 ^{13}C NMR of **4a**

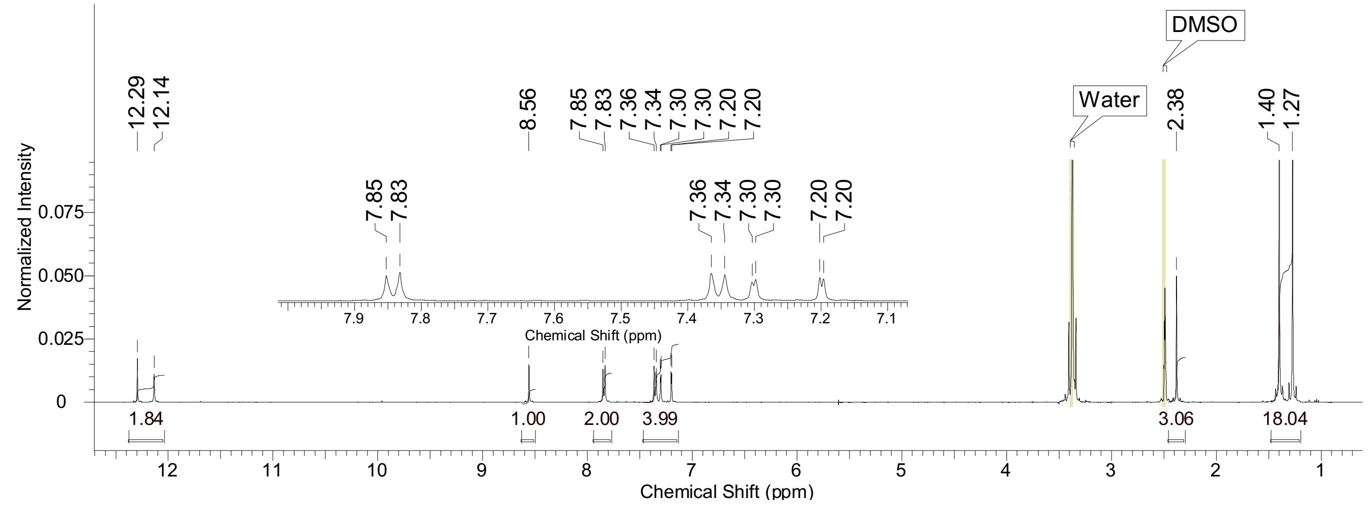

Figure 5 ^{1}H NMR of **4b**

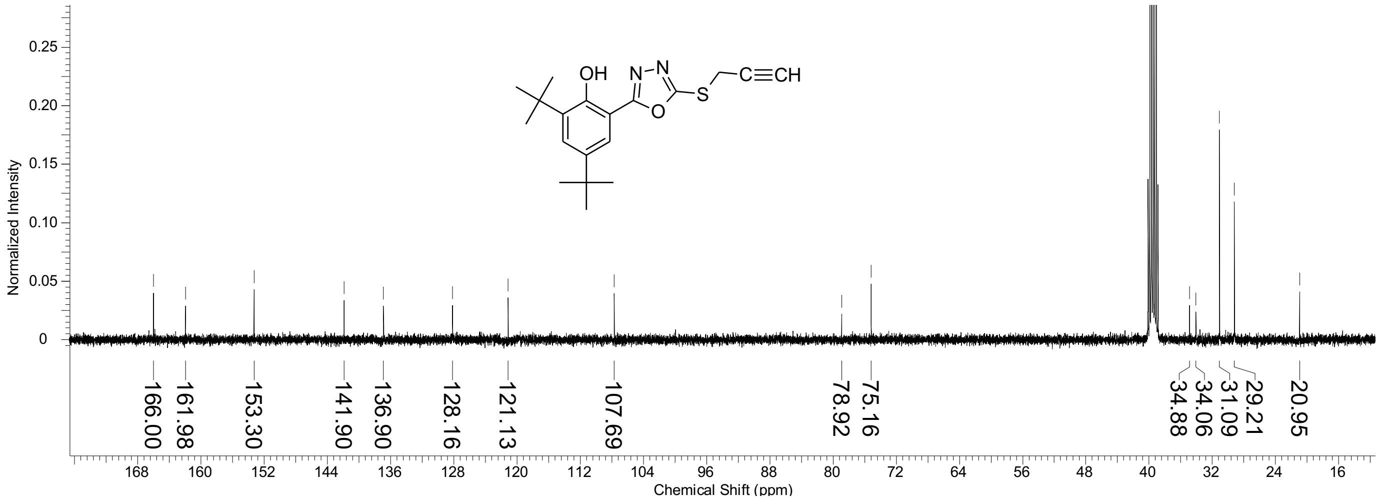

Figure 6 ^{13}C NMR of **4b**

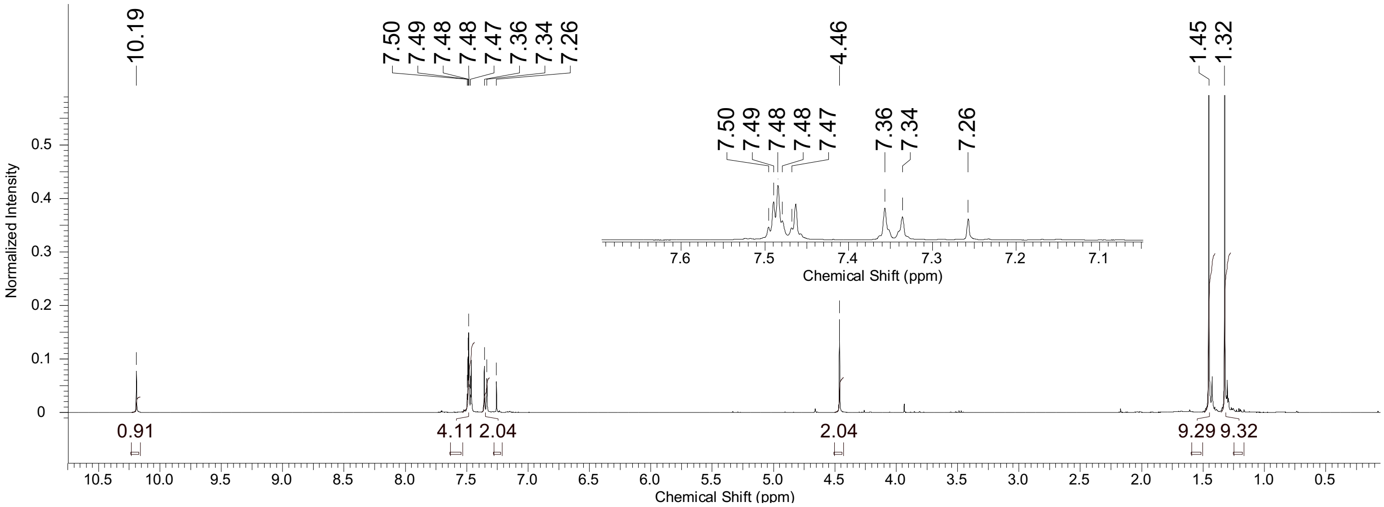

Figure 7 ^{1}H NMR of **4c**

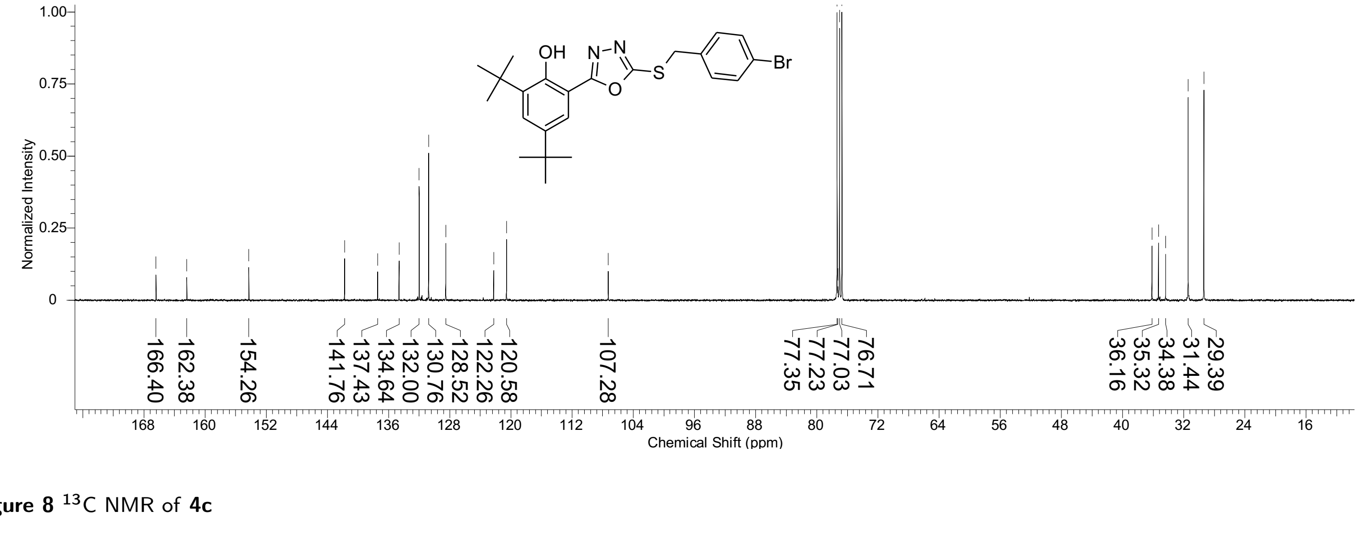

Figure 8 ^{13}C NMR of **4c**

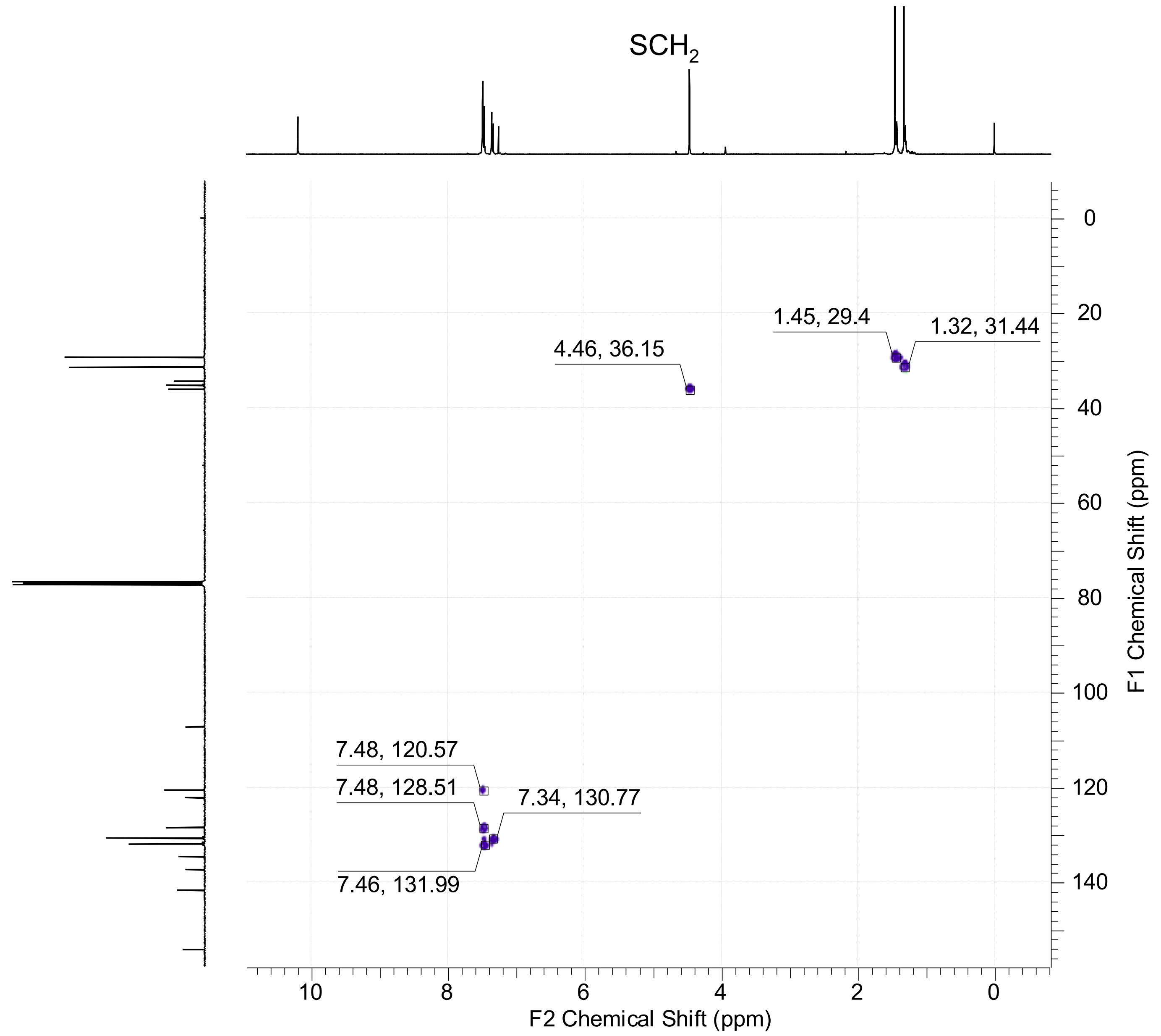

Figure 9 HSQC NMR of **4c**

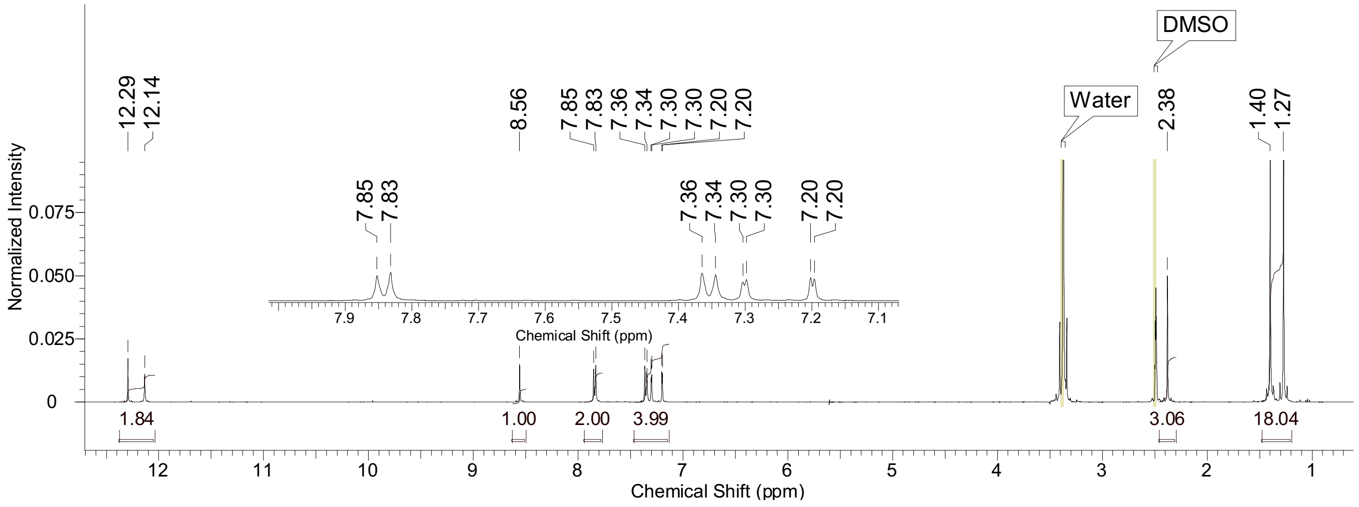

Figure 10 ^{1}H NMR of **5a**

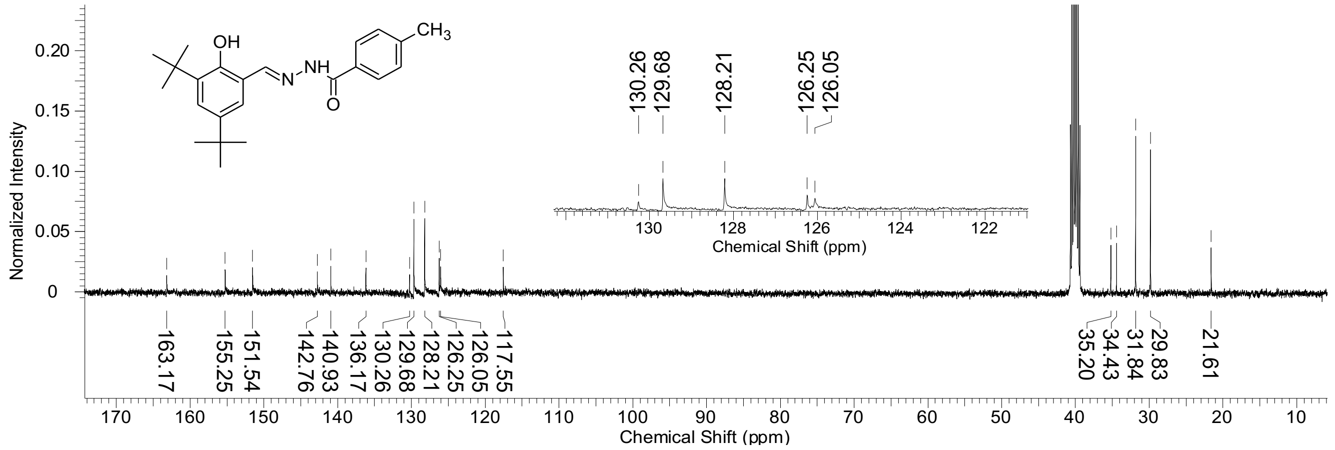

Figure 11 ^{13}C NMR of **5a**

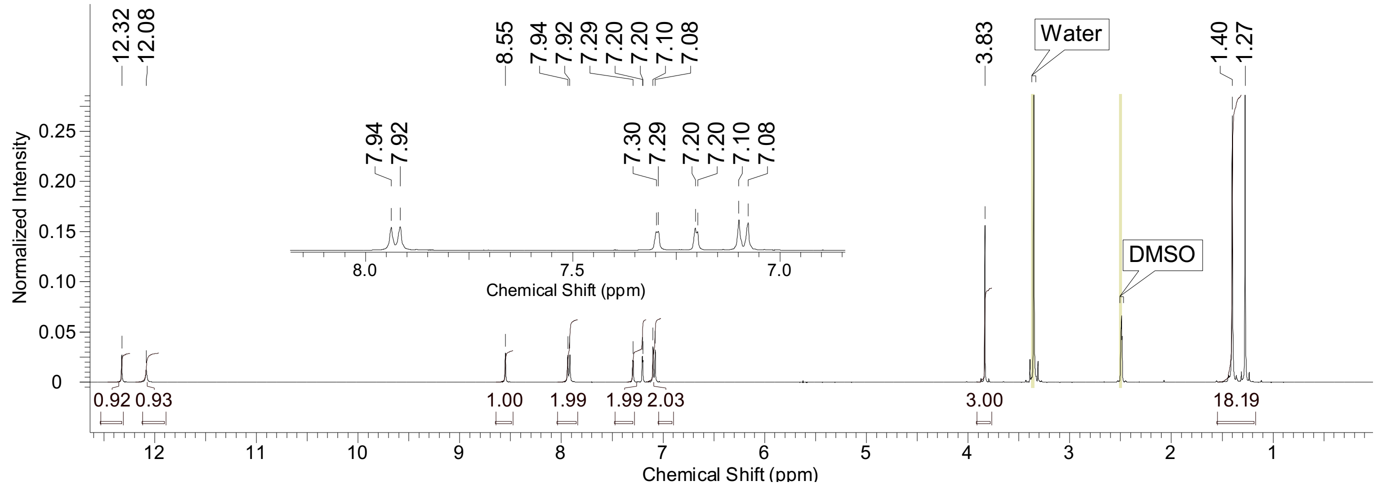

Figure 12 ^{1}H NMR of **5b**

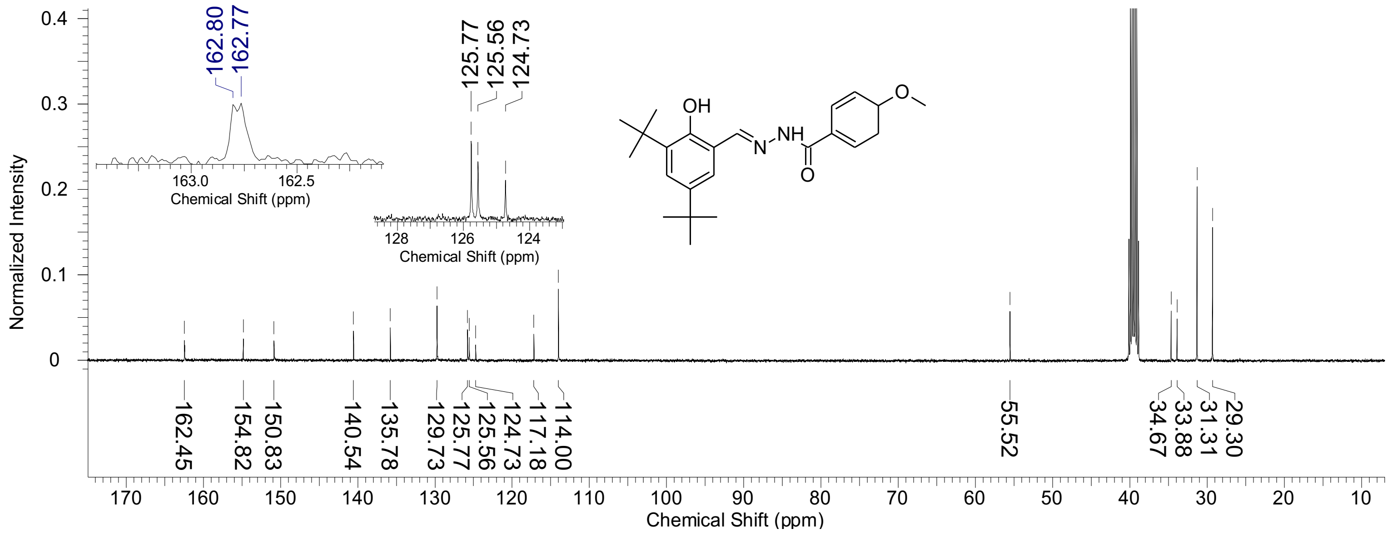

Figure 13 ^{13}C NMR of **5b**

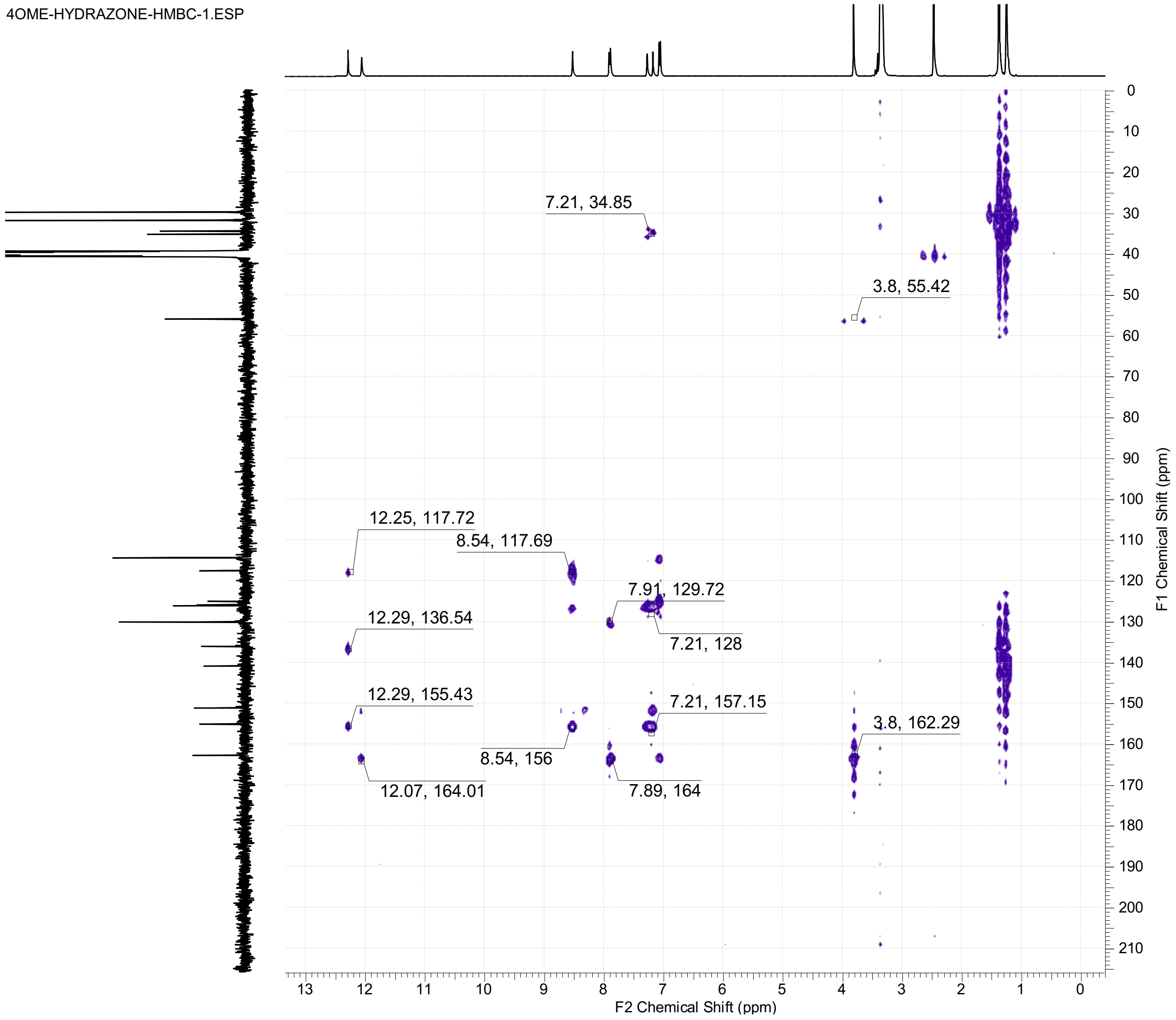

Figure 14 HMBC NMR of **5b**

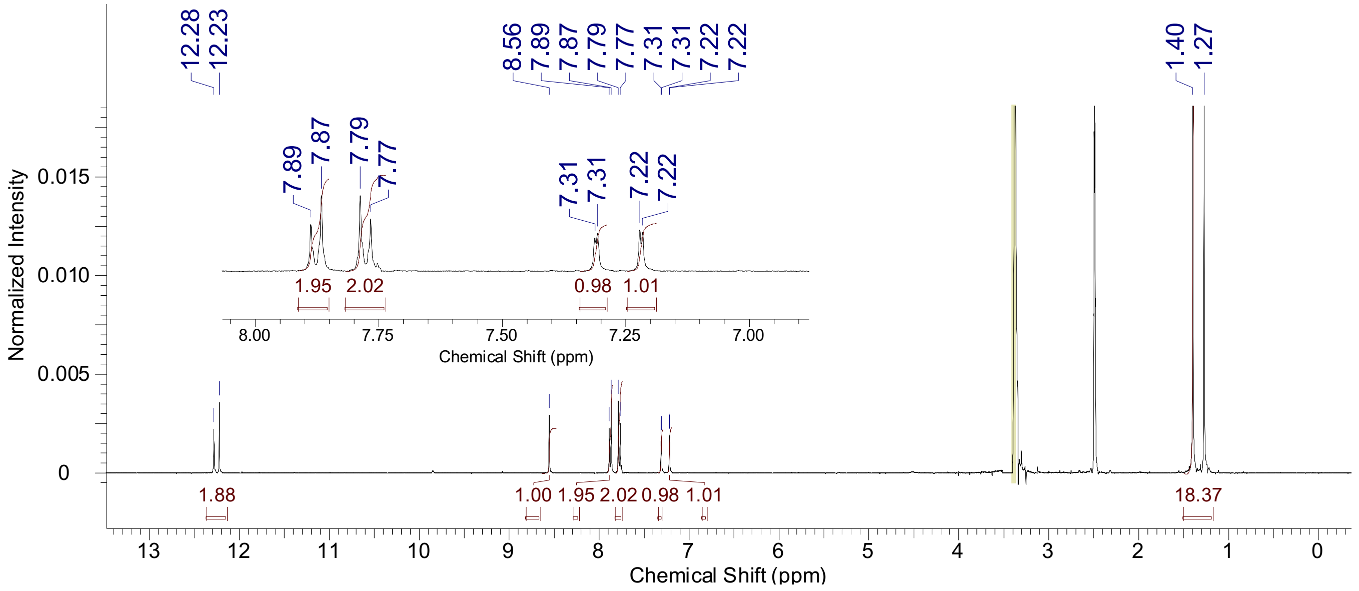

Figure 15 ^{1}H NMR of **5c**

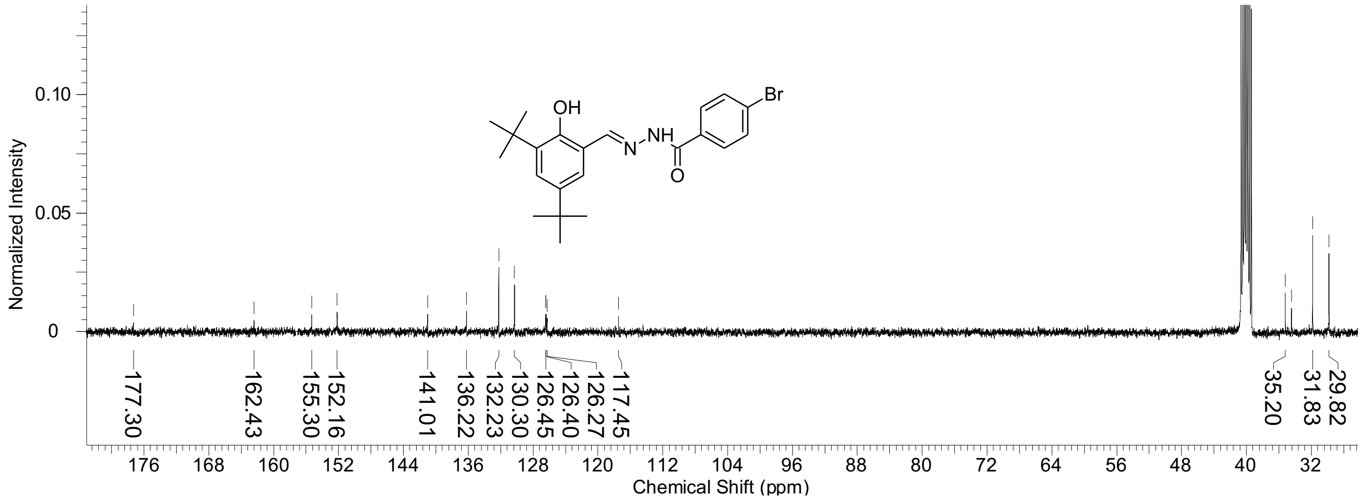

Figure 16 ^{13}C NMR of **5c**

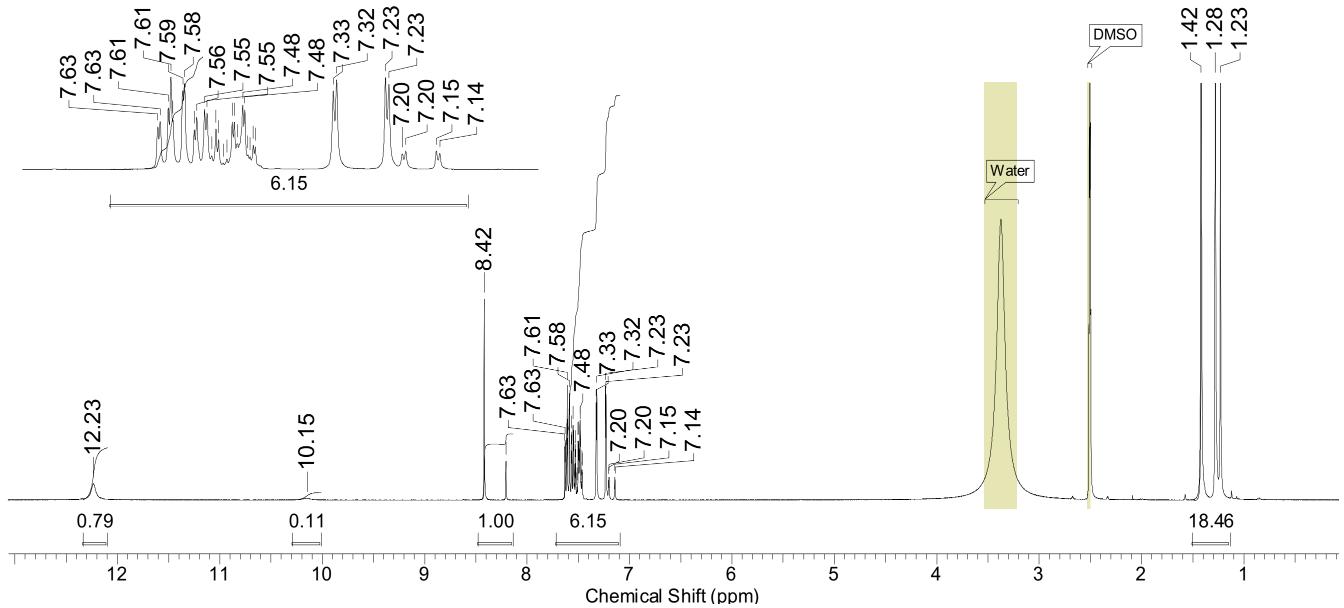

Figure 17 ^{1}H NMR of **5d**

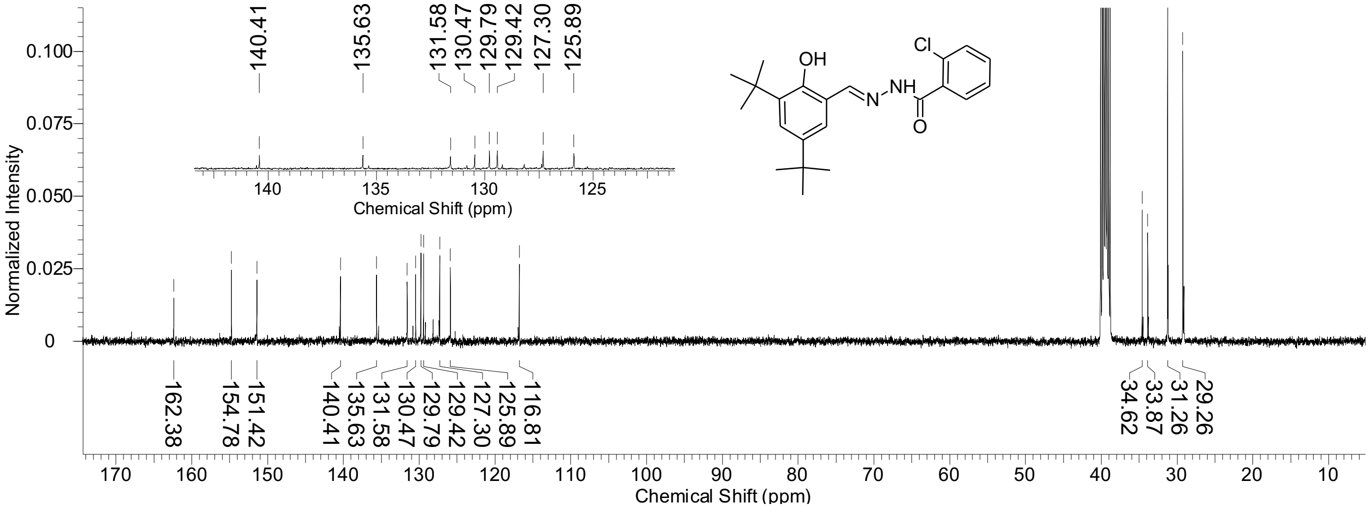

Figure 18 ^{13}C NMR of **5d**

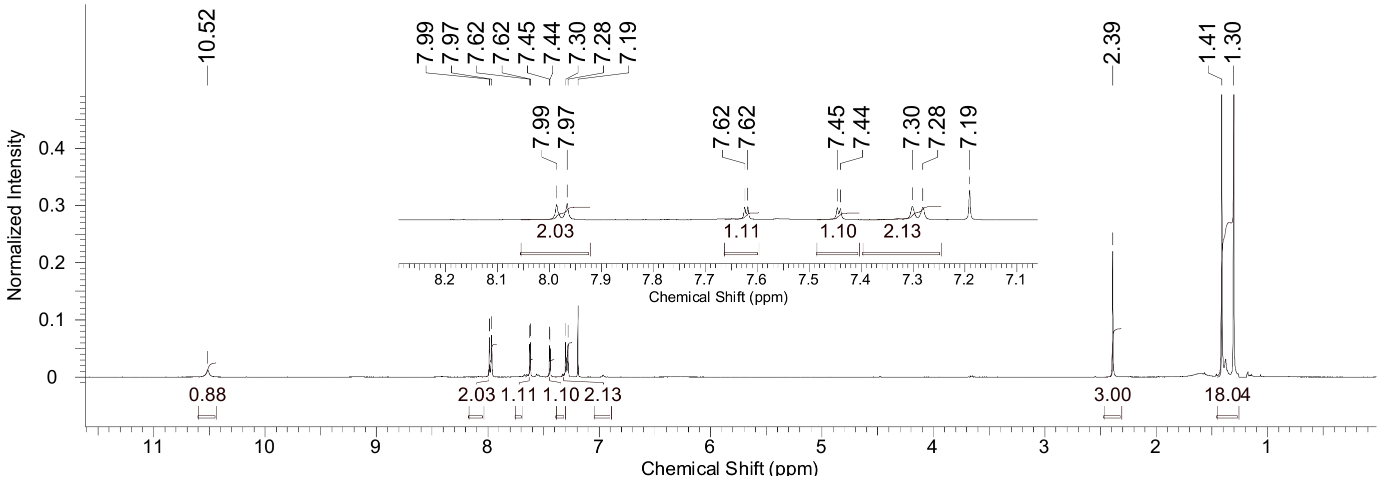

Figure 19 ^{1}H NMR of **6a**

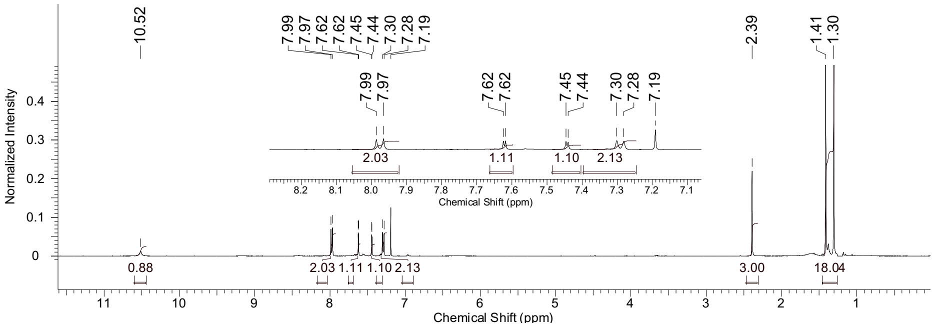

Figure 20 ^{13}C NMR of **6a**

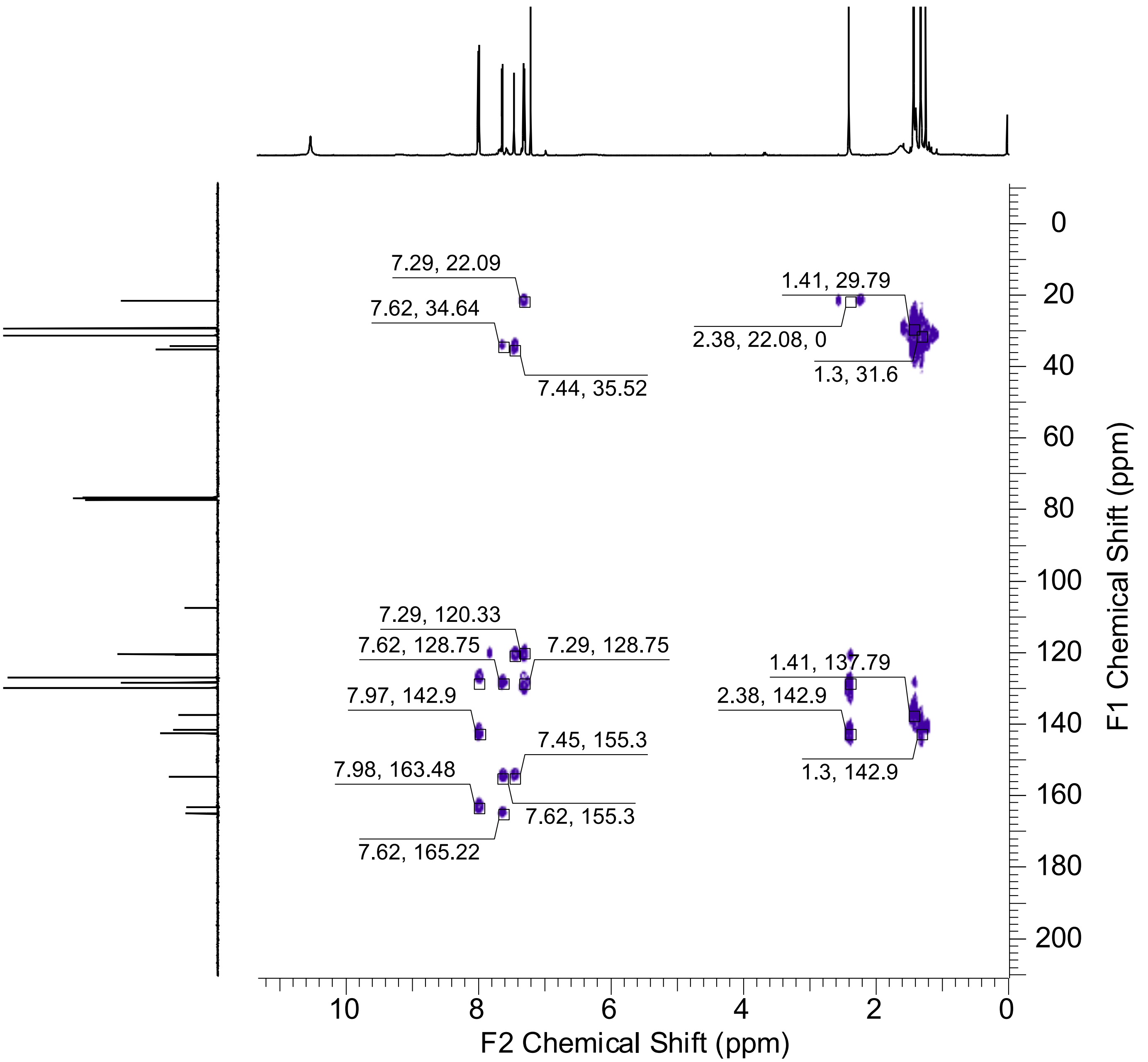

Figure 21 HMBC NMR of **6a**

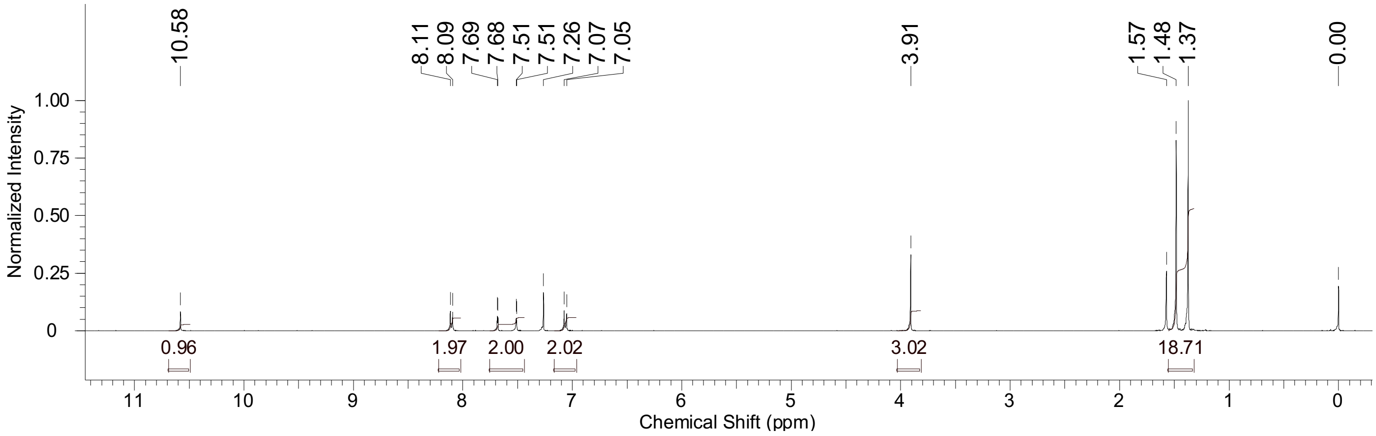

Figure 22 ^{1}H NMR of **6b**

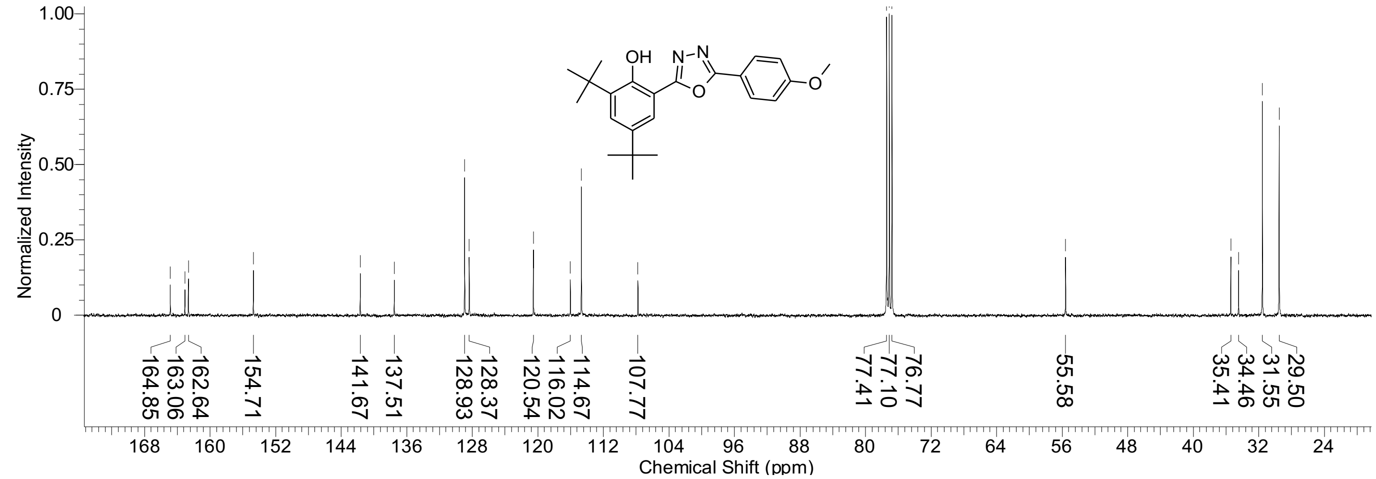

Figure 23 ^{13}C NMR of **6b**

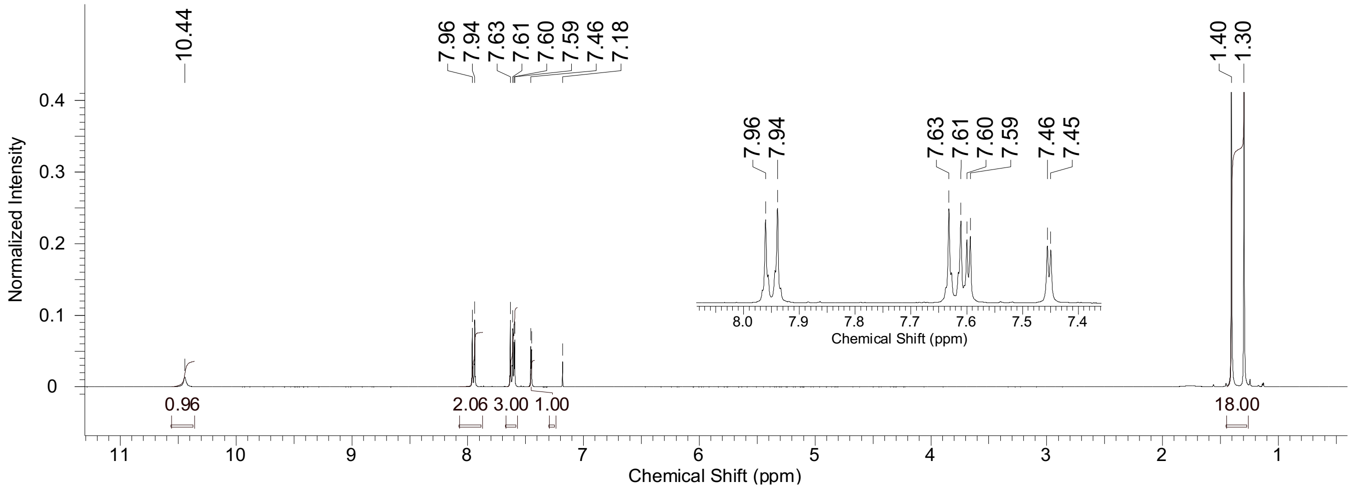

Figure 24 ^{1}H NMR of **6c**

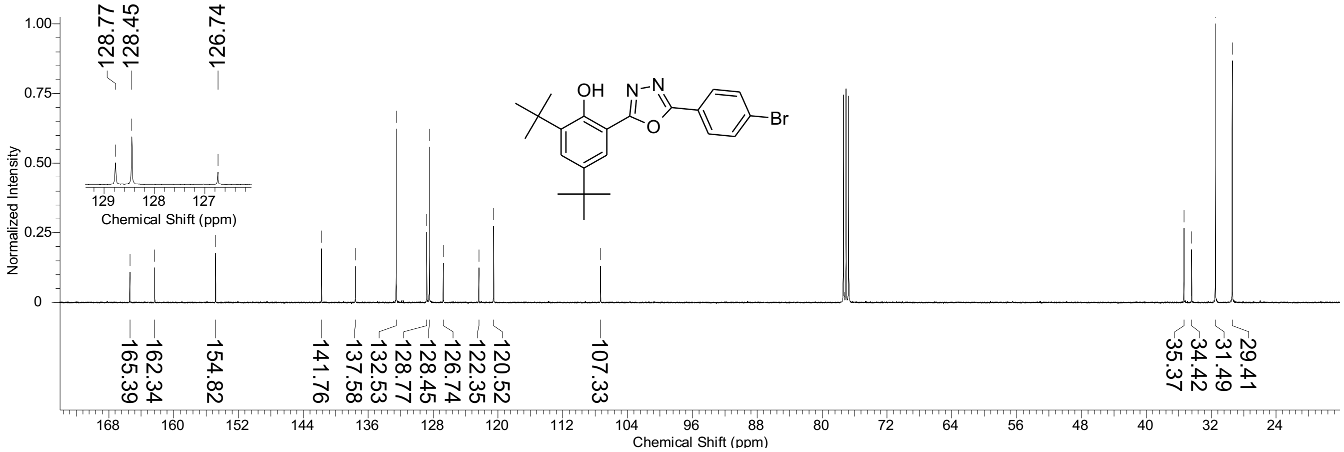

Figure 25 ^{13}C NMR of **6c**

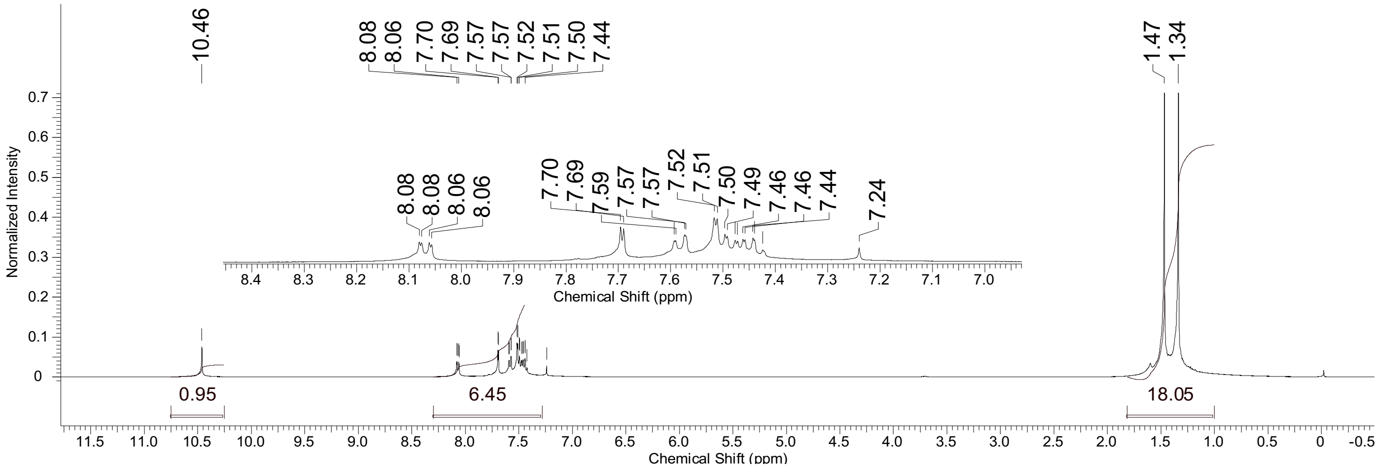

Figure 26 ^{1}H NMR of **6d**

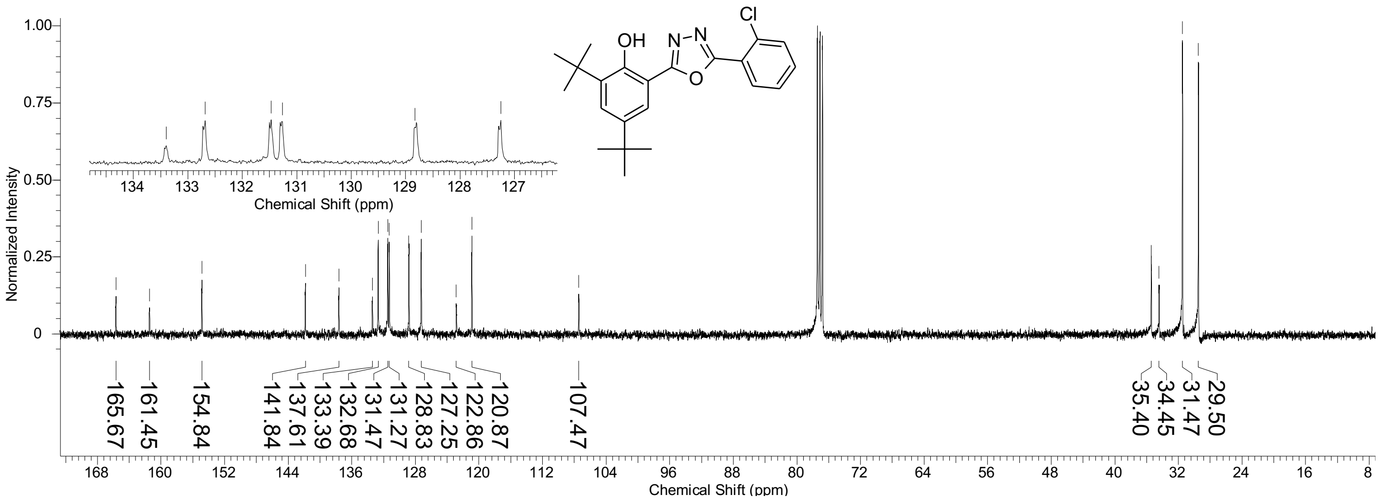

Figure 27 ^{13}C NMR of **6d**

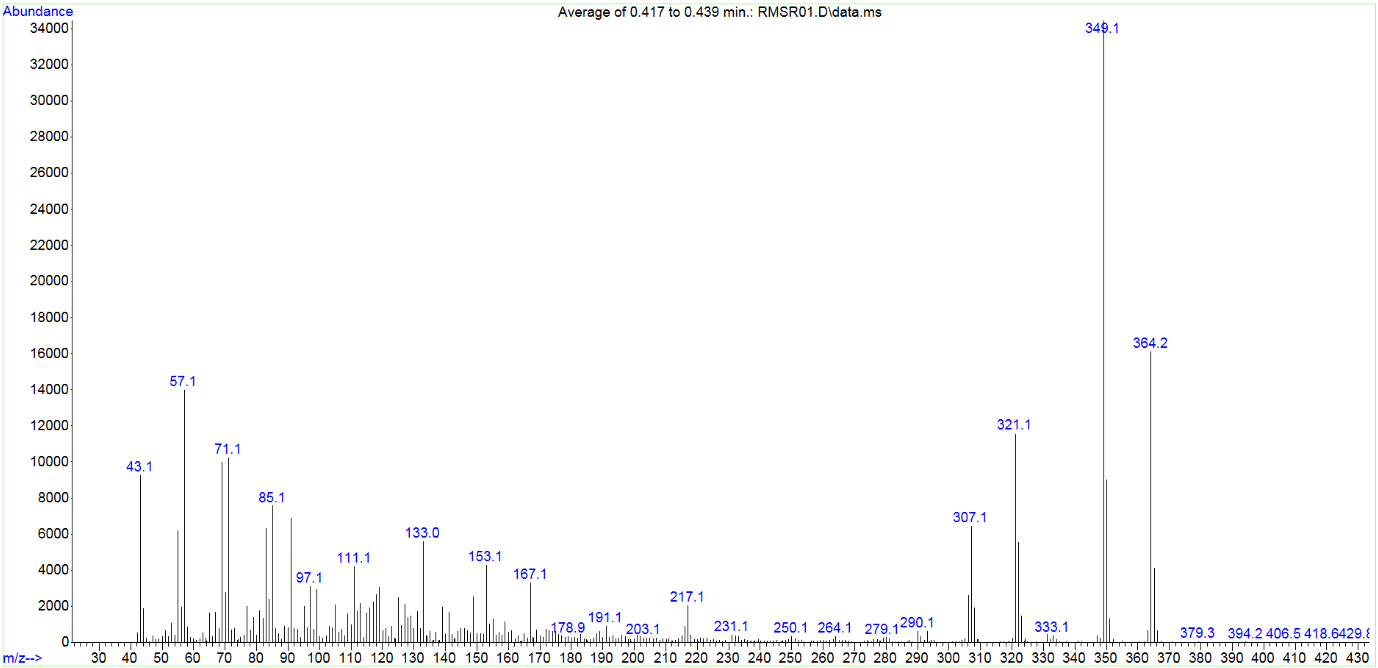

Figure 28 EIMs of **6a**

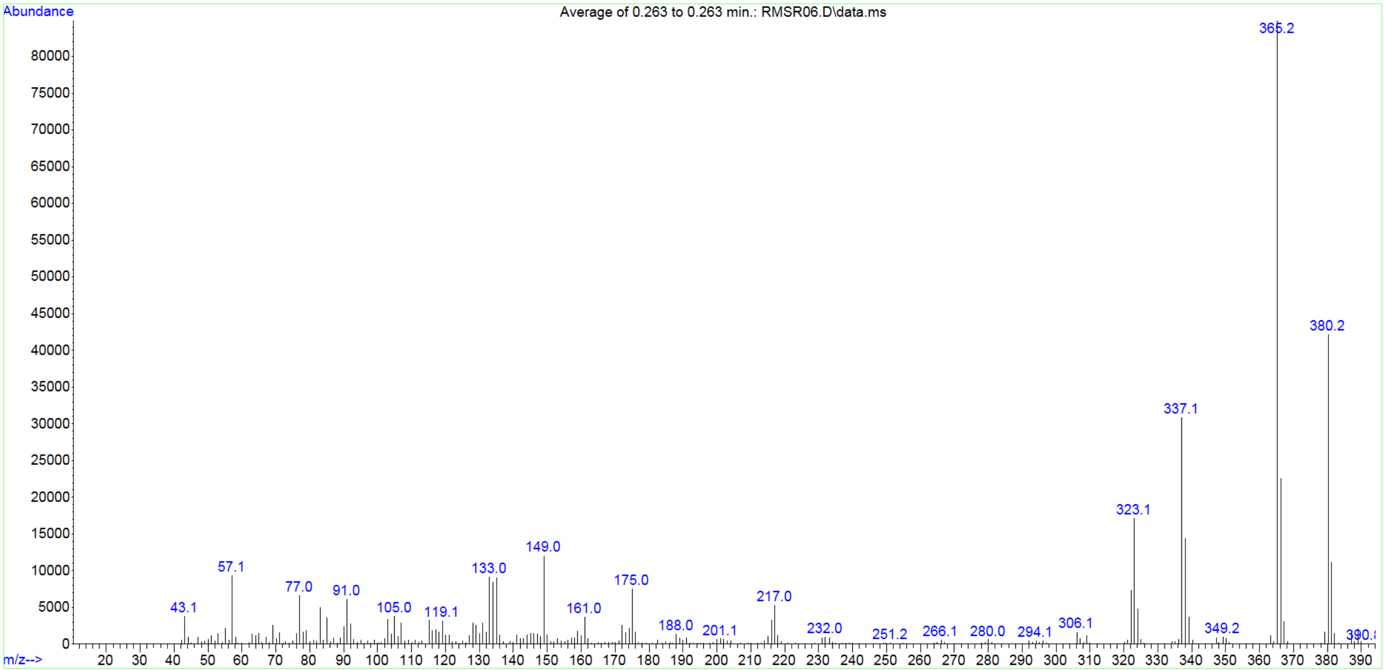

Figure 29 EIMs of **6b**

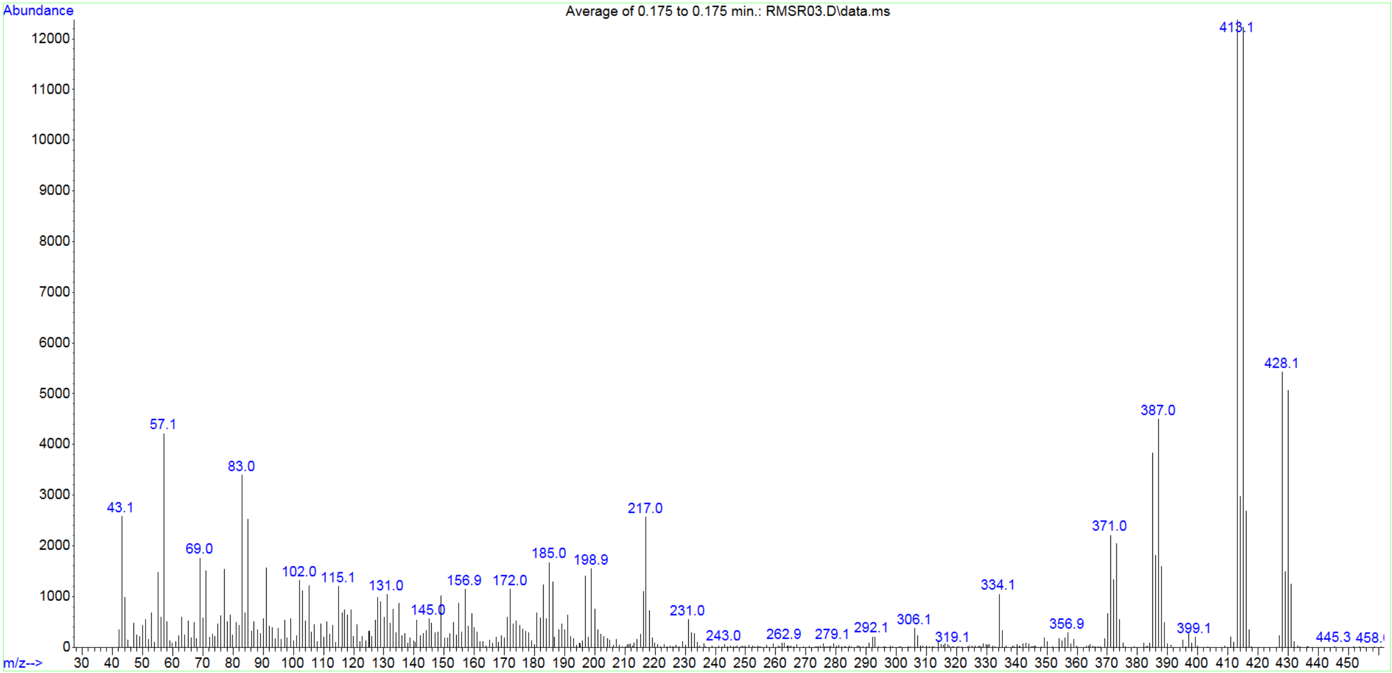

Figure 30 EIMs of **6c**

1.2 The Mass and Loss of HNCO

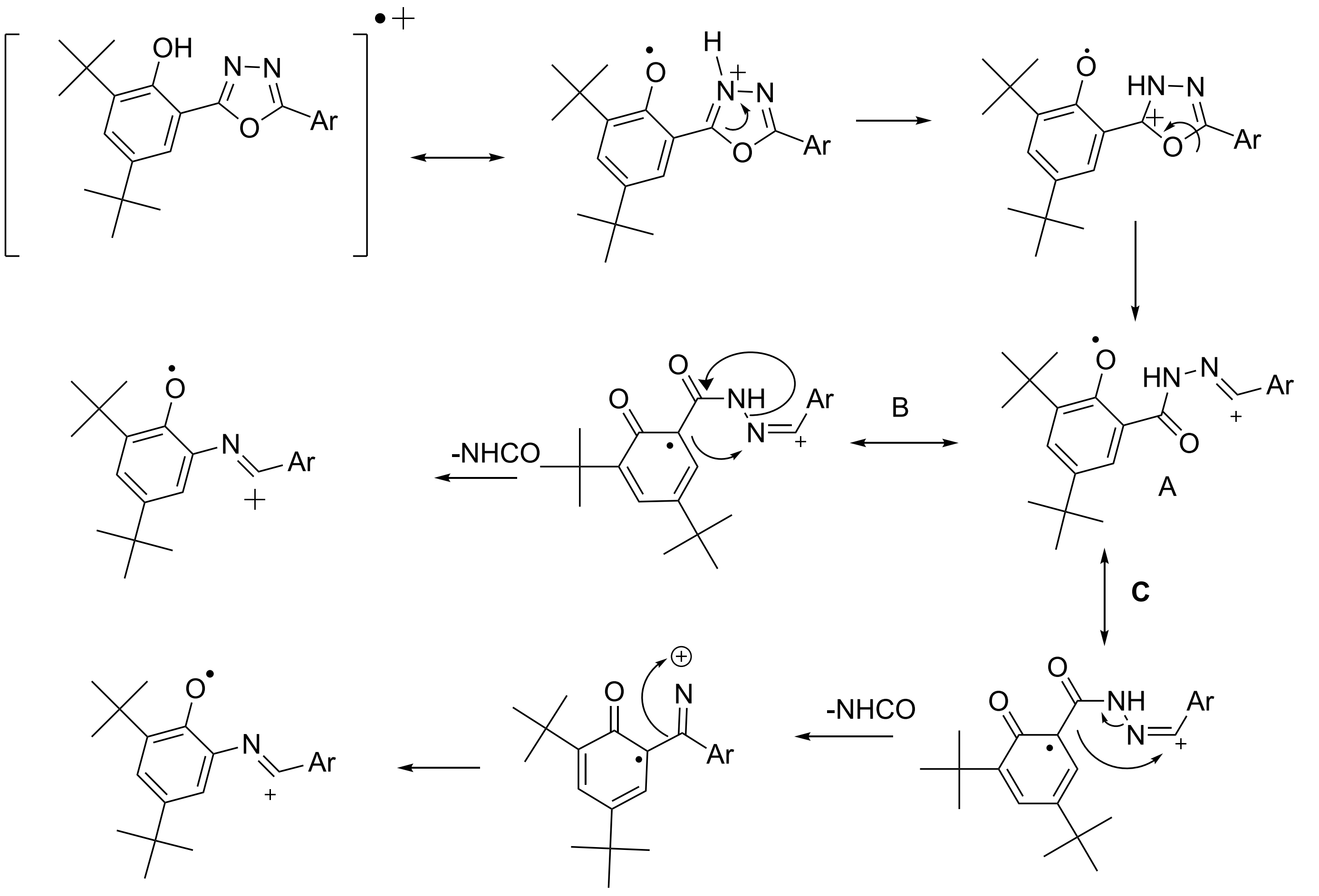

Scheme 1 Proposed pathway of losing HNCO from the 1,3,4-oxadiazole

One interesting fragmentation pattern was the loss of isocyanic acid from 2,5 di-substituted 1,3,4-oxadiazole. The fragmentation pattern has been mentioned in the literature and has been explained as being initiated through M•++H.[1] However, in our study, we observed that the pattern was initiated from M•+, which can be attributed to proton transfer in M•+. This transfer can occur through the rearrangement of molecular ions and migration. The mechanism of losing HNCO is proposed in Scheme 1. The next fragment was also detected ~~(Scheme 2)~~, and EIM spectra are tabulated in Table 1. (Please see Figures 28-30 for the respective EIM spectra.

Compound	*$M^{\bullet+}$*	*%*	*Base peak 100%*	*-HNCO*	*%*	*Next step*	*%*
**6a**	364.2	52	349.1 (-CH_3•)	321.1	32	307.1 (-:CH_2)	20
**6b**	380.2	52	366.1 (-CH_3•)	337.1	35	323.1 (-:CH_2)	19
**6c**	428.1	50	413.1 (-CH_3•)	387.0	41	371.0 (-:CH_2)	20
**6d**	384.1	40	369.1 (-CH_3•)	341.1	32	327.1 (-:CH_2)	15

Table 1 The EI mass for the oxadiazoles and the loss of HNCO value in the next step

2 Supporting Information 2: Theoretical and Computational details

2.1 Optimisation of the Range-Separation Parameter.

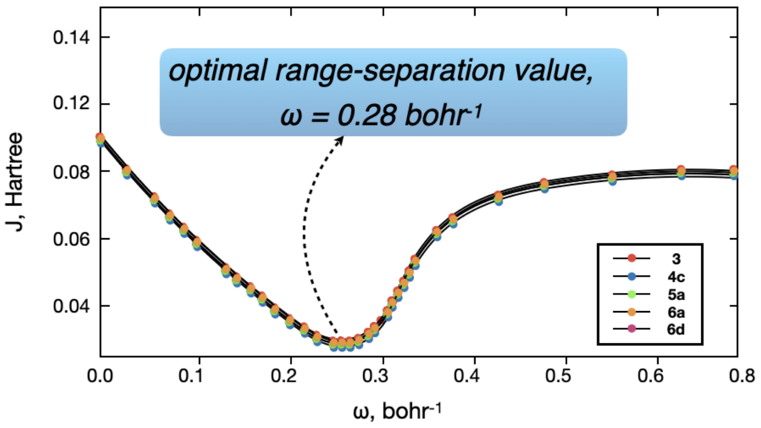

Figure 31 Functions defined in[2] used for optimisation of the range-separation parameter, ω for the selected synthesised compounds.

Certain default values for the range separation parameter were established in[3,4] are used like universal constants in common quantum chemical programs.[5] The value 0.28 bohr[1] was determined by a least-squares fit to empirical data for first- to third-row atoms.[3]

2.2 Radical Scavenging Pathways

Three main mechanisms have been proposed to explain the radical scavenging ability of phenolic antioxidants. Therefore, free radicals can be deactivated by antioxidants according to the following mechanisms.[6–8]

1. Hydrogen atom transfer (HAT, eq 1) from antioxidant molecules (ArOH) to radicals ($R^{\bullet}$)

$$ArOH + R^{\bullet} \rightarrow ArO^{\bullet} + RH \tag{1}$$

2. Two-step reaction: single-electron transfer followed by proton transfer (SET-PT, eq 2)

$$\mathrm{ArOH} + \mathrm{R}^{\bullet} \rightarrow \mathrm{ArOH}^{\bullet+} + \mathrm{R}^{-} \rightarrow RH + \mathrm{ArO}^{\bullet} \tag{2}$$

3. Two-step reaction: sequential proton loss electron transfer (SPLET, eq 3-5)

$$ArOH \rightarrow \mathrm{ArO}^{-} + \mathrm{H}^{+}; \tag{3}$$

$$\mathrm{ArO}^{-} + \mathrm{R}^{\bullet} \rightarrow \mathrm{ArO}^{\bullet}\mathrm{R}^{-}; \tag{4}$$

$$\mathrm{R}^{-} + \mathrm{H}^{+} \rightarrow RH \tag{5}$$

These mechanisms can occur at different periods, at various rates, and with varied priorities. The numerical parameters for BDE, IP, and PDE mechanisms can be computed using theoretical techniques. In the equations below, these parameters are stated.

1.
$$BDE = H^{\bullet}_{ArO} + H^{\bullet}_{H}\breve{}H_{ArO} \tag{6}$$

2.
$$IP = \mathrm{H}_{\mathrm{ArOH}}{}^{\bullet+} + H_{e}\breve{}H_{ArOH} \tag{7}$$

3.
$$PDE = H^{\bullet}_{ArO^{-}} + H_{H^{+}}\breve{}H_{ArOH^{\bullet+}} \tag{8}$$

where H denotes the molecular enthalpy of various species. The BDE parameter (eq 6) can be used to indicate the reactivity of ArOH in the HAT route; the lower the BDE value, the higher the anticipated activity. IP and PDE from $\mathrm{ArOH}^{\bullet+}$ define the SET-PET mechanism. Antioxidants with lower IP and PDE levels are considered to be more active.

Compound 3

0 1

C	0.37990285	-0.17221644	0.51186411
C	1.75655486	0.05256077	0.37685862
C	2.23109408	1.33781003	0.08212343
C	1.32898159	2.39828294	-0.07760206
C	-0.04767017	2.17350644	0.05740691
C	-0.52220964	0.88825648	0.35213863
H	3.28219833	1.50943213	-0.02095770
H	-0.73645316	2.98320074	-0.06454848
C	1.85045320	3.81064456	-0.40148787
C	2.74788753	-1.11279341	0.55238430
C	2.16515020	-2.13763785	1.54319765
H	2.85393324	-2.94733204	1.66515370
H	1.99917972	-1.66507717	2.48873466
H	1.23744874	-2.51257158	1.16412688
C	4.08308398	-0.57316902	1.09796286
H	4.77186709	-1.38286311	1.21991885
H	4.48797284	0.13889832	0.40954067
H	3.91711343	-0.10060845	2.04349986
C	0.79802862	4.56850356	-1.23194088
H	-0.11112266	4.64351973	-0.67273023
H	1.16034973	5.54981984	-1.45697816
H	0.61362872	4.03873550	-2.14311729
C	2.11585143	4.57311473	0.90992471
H	2.84708151	4.04655040	1.48692777
H	2.47817261	5.55443095	0.68488716
H	1.20670023	4.64813112	1.46913548
C	3.15895117	3.70267705	-1.20633323
H	3.89018125	3.17611272	-0.62933014
H	2.97455108	3.17290878	-2.11750952
H	3.52127234	4.68399324	-1.43137078
C	2.98676109	-1.79292753	-0.80848196
H	3.67554418	-2.60262162	-0.68652583
H	2.05905969	-2.16786141	-1.18755276
H	3.39164999	-1.08086028	-1.49690422
C	-2.03501383	0.64124925	0.50049958
C	-4.13927868	0.96261100	0.93191536
N	-3.90852966	-0.30386245	1.05139378
N	-2.55456641	-0.51064231	0.77378354
O	-3.02919757	1.66187231	0.35828408
S	-5.65738246	1.74703341	1.43035543
H	-6.64817738	0.89902423	1.30663871
O	-0.10432122	-1.48369624	0.81260946
H	-0.92000976	-1.41639371	1.31432908

Figure 32 Cartesian coordinates for compound 3.

2.3 Proposed resonance structures of FRAP mechanism

The acidic medium (pH 3.6) is required to facilitate Fe^{3+} complex solubility, yet the condition also results in lower ionisation potential, which promotes the single electron transfer mechanism, as opposed to the hydrogen atom transfer mechanism in DPPH assay (Figure 38).[9]. The activation of the thiol group is caused by electron transfer followed by proton removal from the hydroxyl group.[9] This reaction allows one mole of compound **3** to reduce two moles of Fe^{3+}, resulting in a compound **3** with a high FRAP value and potent antioxidant capability.

Notes and references

[1] B. Frański Rafałand Gierczyk and G. Schroeder, *International Journal of Mass Spectrometry*, 2004, **231**, 47–49.

[2] O. S. Bokareva, G. Grell, S. I. Bokarev and O. Kuhn, *Journal of Chemical Theory and Computation*, 2015, **11**, 1700–1709.

[3] Y. Tawada, T. Tsuneda, S. Yanagisawa, T. Yanai and K. Hirao, *The Journal of Chemical Physics*, 2004, **120**, 8425–8433.

[4] J.-W. Song, T. Hirosawa, T. Tsuneda and K. Hirao, *The Journal of Chemical Physics*, 2007, **126**, 154105.

[5] K. Ishimura, L. McMurchie, E. Davidson and J. Rys, *Journal of Computational Chemistry*, 1993, **14**, 1347–1363.

[6] M. Leopoldini, N. Russo and M. Toscano, *Food Chemistry*, 2011, **125**, 288–306.

[7] J. S. Wright, E. R. Johnson and G. A. DiLabio, *Journal of the American Chemical Society*, 2001, **123**, 1173–1183.

Compound 4c

0 1

C	0.37176817	-0.15992076	0.50846070
C	1.75177602	0.05917033	0.40112330
C	2.23648042	1.33775417	0.09425794
C	1.34120507	2.39724780	-0.10534935
C	-0.03879497	2.17815393	0.00185712
C	-0.52351939	0.89957264	0.30876715
H	3.29014172	1.50503344	0.01236047
H	-0.72236501	2.98709455	-0.15061624
C	1.87389743	3.80229103	-0.44249656
C	2.73567537	-1.10507168	0.62036938
C	2.12763659	-2.11050428	1.61584935
H	2.81127082	-2.91942042	1.76817310
H	1.94130396	-1.62054417	2.54864996
H	1.20786868	-2.49012930	1.22239148
C	4.05945468	-0.55868598	1.18664435
H	4.74309918	-1.36759089	1.33896394
H	4.48191280	0.13989967	0.49497293
H	3.87311836	-0.06872737	2.11944549
C	0.84299693	4.54749445	-1.31055419
H	-0.07859496	4.63474509	-0.77393648
H	1.21312878	5.52372841	-1.54479662
H	0.67818301	4.00178585	-2.21605786
C	2.11110414	4.58771118	0.86075185
H	2.82737866	4.06994260	1.46388440
H	2.48123169	5.56394392	0.62650146
H	1.18951607	4.67496155	1.39737610
C	3.20029343	3.67670396	-1.21482658
H	3.91656301	3.15892902	-0.61169371
H	3.03547641	3.13098960	-2.12032605
H	3.57043038	4.65293349	-1.44907587
C	3.00385012	-1.81024856	-0.72217056
H	3.68748977	-2.61916146	-0.56985867
H	2.08407788	-2.18987771	-1.11561575
H	3.42630997	-1.11166515	-1.41384065
C	-2.04002344	0.65887649	0.42656086
C	-4.15284863	0.99312130	0.80288594
N	-3.92845916	-0.27147088	0.95103907
N	-2.56899076	-0.48654521	0.70884654
O	-3.02776941	1.67906525	0.24258832
S	-5.67997620	1.79022462	1.25109807
O	-0.12283001	-1.46457976	0.82172261
H	-0.94963590	-1.38626912	1.30324694
C	-7.02516486	0.63823992	1.07282707
H	-6.86116666	-0.20340531	1.71285728
H	-7.07975715	0.30815787	0.05647510
C	-8.34644622	1.32798683	1.46019199
C	-8.79580281	1.28659014	2.78694845
C	-9.09945954	1.99705809	0.48590748
C	-9.99818548	1.91426682	3.13941751
H	-8.22085590	0.77573900	3.53083578
C	-10.30183801	2.62472984	0.83836891
H	-8.75635335	2.02866926	-0.52709701
C	-10.75120366	2.58334027	2.16512586
H	-10.34128543	1.88266007	4.15242533
H	-10.87678081	3.13556970	0.09447027
Br	-12.38995558	3.43881088	2.64550839

Figure 33 Cartesian coordinates for compound 4c.

[8] V. Stepanić, K. G. Trošelj, B. Lučić, Z. Marković and D. Amić, *Food chemistry*, 2013, **141**, 1562–1570.

[9] K. A. Wojtunik-Kulesza, *Molecules*, 2020, **25**, 5267.

Compound 5a

0 1

C	0.61663541	0.78115797	-0.48195223
C	1.96396239	0.50298615	-0.21502826
C	2.88232022	1.55260947	-0.07788593
C	2.45337074	2.88041214	-0.20766780
C	1.10605437	3.15859680	-0.47466851
C	0.18768648	2.10896148	-0.61181456
H	3.91103078	1.34021128	0.12592723
H	0.77854293	4.17240468	-0.57381147
C	3.46259584	4.03380766	-0.05686355
C	3.01482177	5.22451373	-0.92479948
H	2.04734717	5.55335691	-0.60738095
H	3.71604962	6.02588850	-0.82001518
H	2.96995480	4.92160717	-1.95004721
C	3.52716994	4.46977182	1.41872845
H	3.83828605	3.64246306	2.02177736
H	4.22839634	5.27114723	1.52351081
H	2.55969463	4.79861405	1.73614606
C	4.85503701	3.56051687	-0.51370285
H	5.16614872	2.73320875	0.08935011
H	4.81017160	3.25760346	-1.53894889
H	5.55626545	4.36188954	-0.40892138
C	2.43538421	-0.95612523	-0.07240805
C	1.28301417	-1.81637810	0.47858637
H	0.45271075	-1.77404630	-0.19498884
H	1.61056724	-2.83017666	0.57768374
H	0.98509237	-1.44261970	1.43589836
C	2.86416644	-1.49406351	-1.45022072
H	3.66483966	-0.89635550	-1.83306254
H	3.19171961	-2.50786179	-1.35111749
H	2.03386009	-1.45173625	-2.12379318
C	3.63040493	-1.01704030	0.89702825
H	3.33248858	-0.64327436	1.85433849
H	3.95796230	-2.03083626	0.99613148
H	4.43107505	-0.41932951	0.51418033
O	-0.32048454	-0.28989016	-0.62182607
H	-0.36080856	-0.56173140	-1.54164925
C	-1.29288185	2.41462194	-0.90523545
H	-1.99408616	1.61319870	-1.00978559
N	-1.68882812	3.64026225	-1.02532757
N	-3.03482535	3.91810155	-1.29196602
H	-3.53163356	4.01821911	-0.42990047
C	-3.13423875	5.16594887	-2.06260769
O	-2.20363318	5.50074770	-2.84071571
C	-4.37722177	6.06350213	-1.91772860
C	-5.41355584	5.69071416	-1.05114618
C	-4.47199446	7.25307397	-2.65247649
C	-6.54463434	6.50753482	-0.91926708
H	-5.34121468	4.78242742	-0.49018045
C	-5.60307475	8.06988752	-2.52060449
H	-3.68074778	7.53768343	-3.31416082
C	-6.63938481	7.69713081	-1.65398361
H	-7.33589180	6.22291583	-0.25759825
H	-5.67543169	8.97815580	-3.08159720
C	-7.88230722	8.59475776	-1.50903899
H	-8.61920949	8.30233459	-2.22762056
H	-8.28454459	8.49048303	-0.52302060
H	-7.60675474	9.61513756	-1.67576343

Figure 34 Electronic energies for compound 5a.

Compound 6a

0 1

C	0.40765527	-0.15898619	0.65029920
C	1.77800431	0.07116658	0.45871297
C	2.22351771	1.32636995	0.02149266
C	1.30070129	2.35196141	-0.21924928
C	-0.06801942	2.12118300	-0.03332624
C	-0.51610580	0.86598356	0.39797009
H	3.26872227	1.50208928	-0.12761006
H	-0.77235703	2.90394240	-0.22079711
C	1.79049355	3.73398414	-0.68753094
C	2.79599909	-1.05350659	0.72766828
C	2.26520552	-1.97085051	1.84477605
H	2.97292374	-2.75147294	2.03182033
H	2.12100554	-1.39902330	2.73762529
H	1.33330169	-2.39948537	1.54106393
C	4.13867817	-0.43686511	1.16390047
H	4.84614742	-1.21786006	1.35008392
H	4.50750748	0.20046285	0.38737835
H	3.99562389	0.13494612	2.05672936
C	0.70217621	4.39825460	-1.55073034
H	-0.19072508	4.51320931	-0.97238045
H	1.04226734	5.35905210	-1.87638410
H	0.49857307	3.78426808	-2.40292049
C	2.08291285	4.61683776	0.53976665
H	2.83914919	4.15462753	1.13915829
H	2.42283198	5.57811037	0.21531936
H	1.18971823	4.73098530	1.11790926
C	3.07551524	3.56873517	-1.51991644
H	3.83186303	3.10696261	-0.92058516
H	2.87173995	2.95500245	-2.37248566
H	3.41543288	4.52965989	-1.84539962
C	3.00338149	-1.87686907	-0.55692753
H	3.71190541	-2.65651978	-0.37011936
H	2.07129687	-2.30648313	-0.85914207
H	3.37103605	-1.23973784	-1.33416797
C	-2.02465984	0.61873673	0.59067227
C	-4.13390480	0.96969465	0.98481671
N	-3.88804774	-0.26973339	1.26307000
N	-2.53189283	-0.49617794	1.00650710
O	-3.02936847	1.60503184	0.33259208
O	-0.04736569	-1.43798013	1.10042907
H	-0.85490596	-1.33008382	1.60904159
C	-5.45690195	1.68656362	1.31705998
C	-5.62473647	3.03478156	0.97262904
C	-6.49152925	0.99429381	1.96052686
C	-6.82719816	3.69029754	1.26779791
H	-4.83357290	3.56359488	0.48340760
C	-7.69530848	1.64973777	2.25503275
H	-6.36267883	-0.03445476	2.22652970
C	-7.86285798	2.99784437	1.90898371
H	-6.95421404	4.71950744	1.00364305
H	-8.48680652	1.12063134	2.74429003
C	-9.18506506	3.71775089	2.23317968
H	-9.08399616	4.25111021	3.15524959
H	-9.41888271	4.40519823	1.44727545
H	-9.97099231	2.99713878	2.32226696

Figure 35 Electronic energies for compound 6a.

Compound 6d

0 1

C	0.39531216	-0.14482330	0.62256382
C	1.76835553	0.08054436	0.45571276
C	2.22556661	1.33330968	0.02508469
C	1.30971052	2.36067632	-0.23873525
C	-0.06334033	2.13529364	-0.07184004
C	-0.52050980	0.88256302	0.35885077
H	3.27393423	1.50541063	-0.10225127
H	-0.76262908	2.91971662	-0.27324163
C	1.81206410	3.73732572	-0.71204202
C	2.77470739	-1.04850305	0.74554168
C	2.20997503	-1.97053844	1.84213504
H	2.90920279	-2.75505384	2.04347124
H	2.04356563	-1.40347346	2.73412709
H	1.28478990	-2.39373094	1.51073432
C	4.10625915	-0.43938241	1.22258179
H	4.80550161	-1.22387341	1.42394168
H	4.49862440	0.20126983	0.46068051
H	3.93981352	0.12768022	2.11457375
C	0.74591074	4.39172098	-1.61017995
H	-0.16106867	4.51418561	-1.05582848
H	1.09486159	5.34827130	-1.93902500
H	0.56314993	3.76737338	-2.45970694
C	2.07513692	4.63594521	0.51059519
H	2.81590459	4.18126942	1.13463755
H	2.42412111	5.59247801	0.18170873
H	1.16816432	4.75845748	1.06493221
C	3.11746765	3.56094749	-1.50984780
H	3.85819835	3.10620270	-0.88578887
H	2.93469546	2.93659835	-2.35937791
H	3.46650996	4.51747274	-1.83869351
C	3.01420310	-1.86462499	-0.53823425
H	3.71340270	-2.64915216	-0.33691154
H	2.08899749	-2.28780759	-0.86964701
H	3.40657808	-1.22396928	-1.30012929
C	-2.02926035	0.63493844	0.54236642
C	-4.13472135	0.97559919	0.95216667
N	-3.88788650	-0.26855449	1.20255235
N	-2.53310567	-0.48777519	0.93868789
O	-3.03662619	1.62329291	0.30028755
O	-0.07125676	-1.42317568	1.06189465
H	-0.88502355	-1.31320404	1.55919893
C	-5.45375744	1.68077667	1.31879732
C	-5.63311078	3.03596764	1.01019463
C	-6.47469980	0.96729896	1.96107843
C	-6.83338568	3.67770707	1.34397451
C	-7.67497568	1.60905613	2.29489011
H	-6.33779727	-0.06744698	2.19661559
C	-7.85430566	2.96427017	1.98635525
H	-6.97030166	4.71240873	1.10837103
H	-8.45447721	1.06433673	2.78534701
H	-8.77072232	3.45425941	2.24124908
Cl	-4.35095000	3.93192622	0.20337649

Figure 36 Electronic energies for compound 6d.

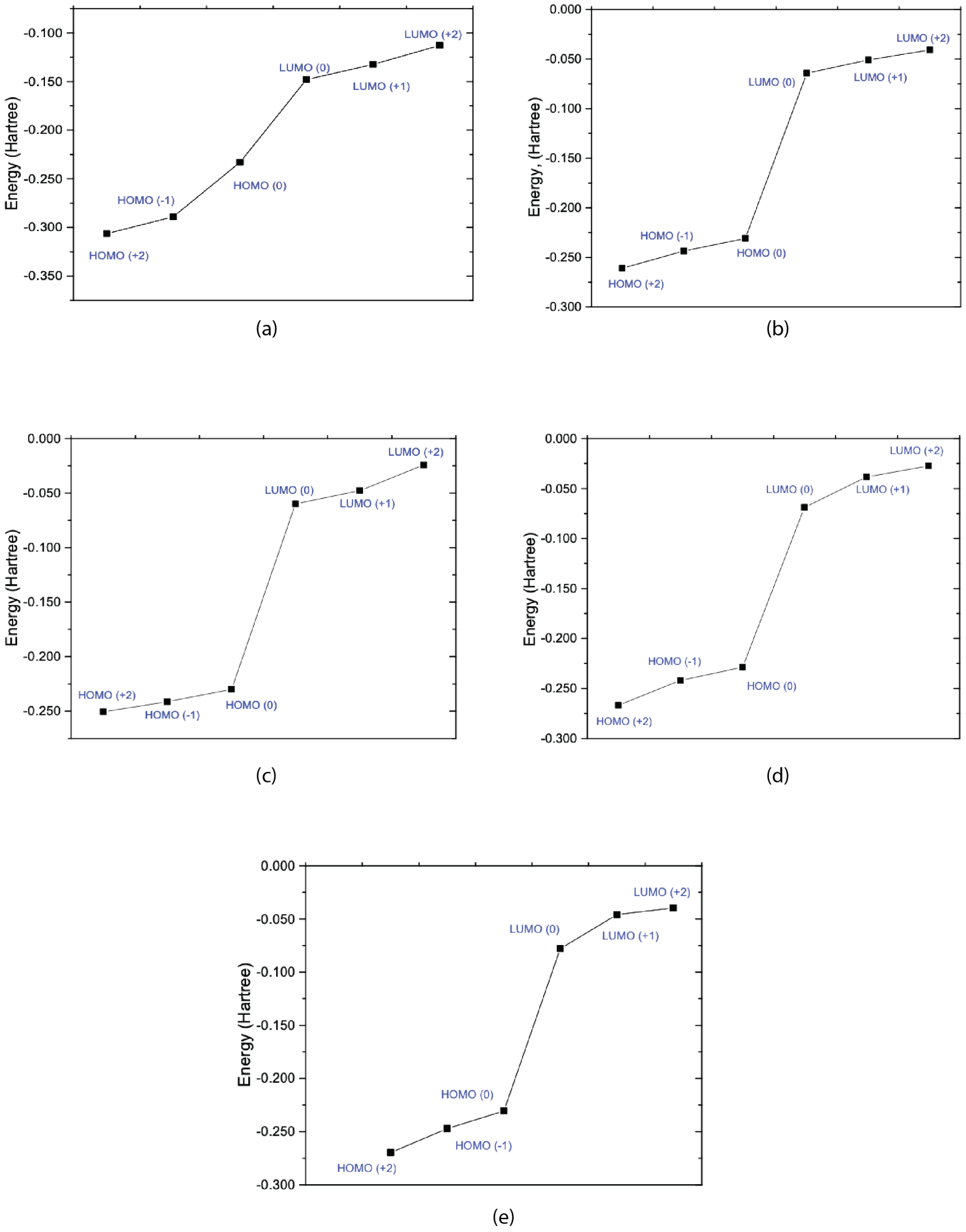

Figure 37 Electronic energies for compounds (a) 3; (b) 4c; (c) 5a; (d) 6a and (e) 6d in the gas phase at B3lyp/6-311++g(d,p).

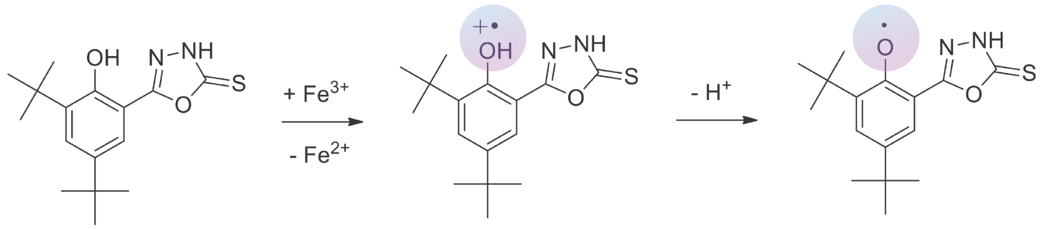

3

Figure 38 Single Electron Transfer (SET) Mechanism of Compound **3**.
